# Illumination of Murine Gammaherpesvirus-68 Cycle Reveals a Sexual Transmission Route from Females to Males in Laboratory Mice

**DOI:** 10.1371/journal.ppat.1003292

**Published:** 2013-04-04

**Authors:** Sylvie François, Sarah Vidick, Mickaël Sarlet, Daniel Desmecht, Pierre Drion, Philip G. Stevenson, Alain Vanderplasschen, Laurent Gillet

**Affiliations:** 1 Immunology-Vaccinology (B43b), Department of Infectious and Parasitic Diseases, Faculty of Veterinary Medicine, University of Liège, Liège, Belgium; 2 Pathology (B43), Department of Morphology and Pathology, Faculty of Veterinary Medicine, University of Liège, Liège, Belgium; 3 Animal Facility (B23), GIGA-University of Liège, Liège, Belgium; 4 Division of Virology, Department of Pathology, University of Cambridge, Cambridge, United Kingdom; Emory University, United States of America

## Abstract

Transmission is a matter of life or death for pathogen lineages and can therefore be considered as the main motor of their evolution. Gammaherpesviruses are archetypal pathogenic persistent viruses which have evolved to be transmitted in presence of specific immune response. Identifying their mode of transmission and their mechanisms of immune evasion is therefore essential to develop prophylactic and therapeutic strategies against these infections. As the known human gammaherpesviruses, Epstein-Barr virus and Kaposi's Sarcoma-associated Herpesvirus are host-specific and lack a convenient *in vivo* infection model; related animal gammaherpesviruses, such as murine gammaherpesvirus-68 (MHV-68), are commonly used as general models of gammaherpesvirus infections *in vivo*. To date, it has however never been possible to monitor viral excretion or virus transmission of MHV-68 in laboratory mice population. In this study, we have used MHV-68 associated with global luciferase imaging to investigate potential excretion sites of this virus in laboratory mice. This allowed us to identify a genital excretion site of MHV-68 following intranasal infection and latency establishment in female mice. This excretion occurred at the external border of the vagina and was dependent on the presence of estrogens. However, MHV-68 vaginal excretion was not associated with vertical transmission to the litter or with horizontal transmission to female mice. In contrast, we observed efficient virus transmission to naïve males after sexual contact. *In vivo* imaging allowed us to show that MHV-68 firstly replicated in penis epithelium and *corpus cavernosum* before spreading to draining lymph nodes and spleen. All together, those results revealed the first experimental transmission model for MHV-68 in laboratory mice. In the future, this model could help us to better understand the biology of gammaherpesviruses and could also allow the development of strategies that could prevent the spread of these viruses in natural populations.

## Author Summary

Epstein-Barr virus and the Kaposi's Sarcoma-associated Herpesvirus are two human gammaherpesviruses which are linked to the development of several cancers. Efficient control of these infections is therefore of major interest, particularly in some epidemiological circumstances. These viruses are however host-specific and cannot be experimentally studied *in vivo*. The identification of a closely related viral species, called Murid herpesvirus 4 with the main strain called murine gammaherpesvirus-68 (MHV-68), in wild rodents opened new horizons to the study of gammaherpesvirus biology. Surprisingly, despite 30 years of research, MHV-68 transmission had never been observed in captivity. In this study, using *in vivo* imaging, we showed that MHV-68 is genitally excreted after latency establishment in intranasally infected female mice. This allowed us to observe, for the first time, sexual transmission of MHV-68 between laboratory mice. In the future, this model should be important to better understand the biology of gammaherpesviruses and should also allow the development of strategies that could prevent the spread of these viruses in natural populations.

## Introduction

Herpesviruses are important pathogens which are ubiquitous in both human and animal populations. They establish persistent, productive infections, with virus carriers both making anti-viral immune responses that protect against disease and excreting infectious virions. Among herpesviruses, gammaherpesviruses establish a long-term latent infection of circulating lymphocytes. They drive lymphocyte proliferation as part of normal host colonization and consequently they can induce some lymphoproliferative disorders. In humans, Epstein-Barr virus (EBV) and the Kaposi's Sarcoma-associated Herpesvirus (KSHV) are associated with several human malignancies such as Burkitt's and Hodgkin's lymphomas, nasopharyngeal carcinoma, Kaposi's sarcoma and post-transplant lymphoproliferative disease [1], [2]. Human cancers associated with these two viruses are particularly prevalent in Africa where they are linked to malaria [3] and human immunodeficiency virus-1 (HIV-1) infection [4]. More generally, individuals with inherited or acquired immunodeficiency have an increased risk of developing a malignancy caused by one of these two viruses [5]. Efficient control of these infections is therefore of major interest, particularly in some epidemiological circumstances.

Knowledge and understanding of the mechanisms of virus transmission in populations are essential to implement large scale antiviral strategies. EBV is mainly shed from the oropharynx into saliva for horizontal spread of the infection to new hosts through mouth-to-mouth contact [6]–[8]. Similarly, horizontal transmission by saliva appears the most common route of KSHV spread in a population [9]. However, several studies in the past decades pointed to human gammaherpesviruses shedding through other routes such as the uterine cervix [10]–[13] or male genital tract [14], [15]. Thus, EBV and KSHV transmission could be more complex than previously thought.

Experimental studies are difficult to perform directly with human gammaherpesviruses because they show limited lytic growth *in vitro* and have no well-established *in vivo* infection model. However, the identification of a closely related virus, murine gammaherpesvirus-68 (MHV-68), in wild rodents offered the possibility of developing a mouse model of gammaherpesvirus pathogenesis [16]. MHV-68 readily infects laboratory mouse (*Mus musculus*) which is a valuable model for *in vivo* studies [17]. Experimental MHV-68 infection typically employs intranasal virus inoculation under general anaesthesia. This leads to a lytic infection of nose and of lung alveolar epithelial cells that is controlled within 2 weeks [18]. Virus meanwhile seeds to lymphoid tissue, mainly draining lymph nodes and spleen [19], and drives the proliferation of latently infected B cells. This peaks at 2 weeks post-infection (p.i.) and is controlled by 4 weeks. A predominantly latent infection of memory B cells then persists for life [20]–[22]. Macrophages and dendritic cells (DCs) also harbour latent MHV-68 infection [20].

Although MHV-68 has been studied for more than 30 years [16], attempts to demonstrate horizontal transmission in laboratory mice have been almost entirely unsuccessful [20], [21]. The only description of horizontal transmission of MHV-68 occurred in two uninfected mouse mothers which had eaten their diseased offspring previously inoculated with the virus [23]. This limited description leaves therefore unresolved how MHV-68 is spread in wild rodent hosts [20], [21]. Different hypotheses can be mounted to explain these poor results. Firstly, conventional animal caging could not allow physiological behaviours observed in the wild such as scent-marking or male fighting. Secondly, although the MHV-68 life cycle in mice following experimental infection is considered as well-known, unexplored inoculation routes could lead to important differences.

Methods available to follow viral infections are constantly evolving, becoming more sensitive and efficient. Recently, a bioluminescence imaging technique has been developed to measure the activity of luciferase reporters in living mice noninvasively and repetitively [24]. This technique has been successfully applied to MHV-68 [17], [19], [25]. In this study, we pursued this work. This allowed us to detect infectious virus in the genital tract of female mice after the time of latency establishment. This presence of infectious virus in the genital tract of latently infected females was transient and under the dependance of sexual steroid hormones. Strikingly, presence of infectious virus in female genital tract allowed us to observe sexual transmission of MHV-68 to naïve males.

## Results

### MHV-68 reaches female genital tract after intranasal infection

**Figure 1:**
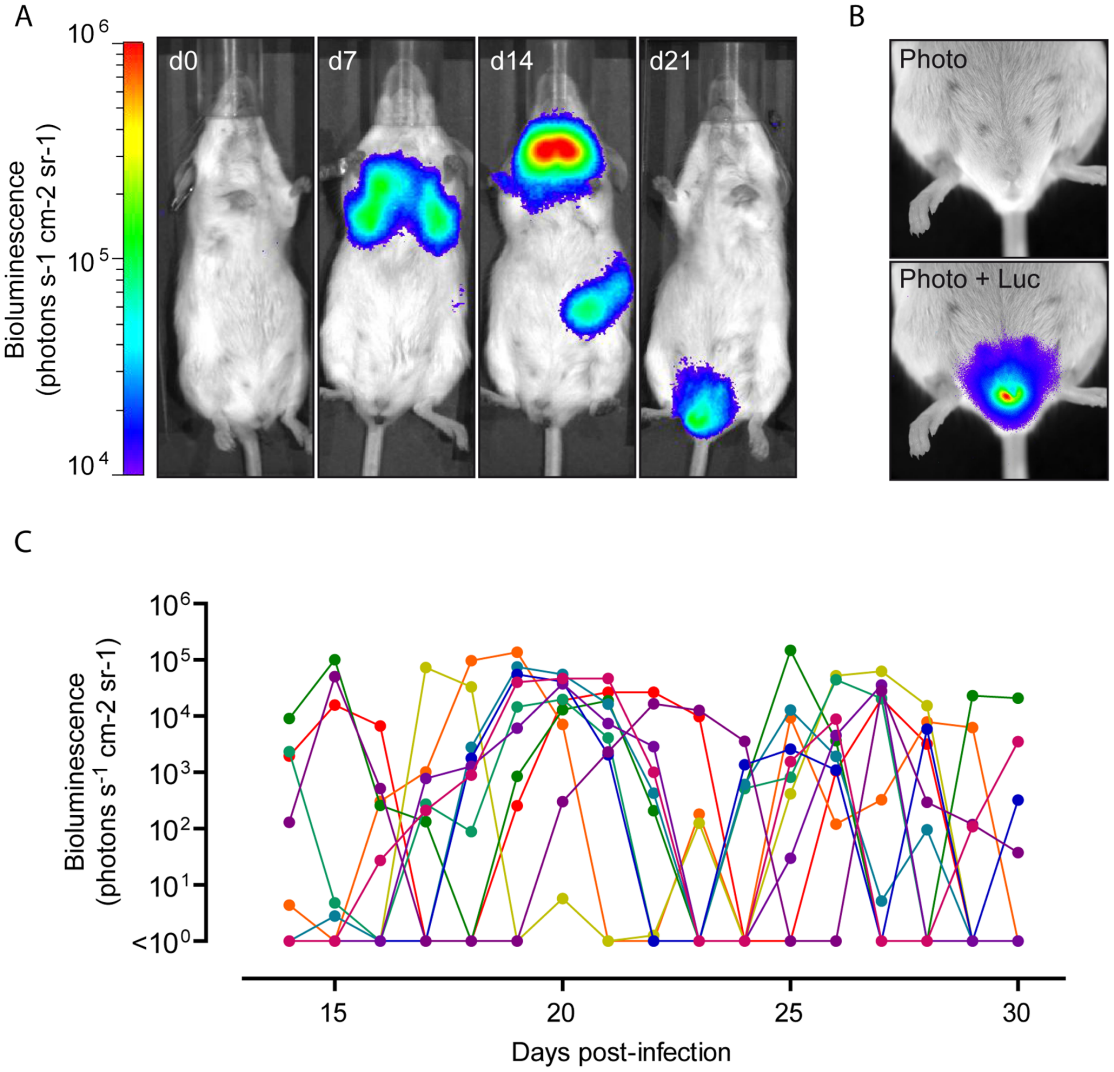
*In vivo* infection by luciferase-expressing MHV-68. Female mice were infected intranasally (10^4^ PFU) with WT luciferase^+^ MHV-68 under general anaesthesia, and then injected with luciferin and imaged every days. **A.** Images show a representative mouse at days 0, 7, 14 and 21 post-infection (p.i.). **B.** Specific signal from the genital region was highlighted in an equivalent mouse. The scale bar (photons sec^−1^ cm^−2^ steradian^−1^) shows the color scheme for signal intensity. The same scale bar is used in A and B. **C.** Temporal progression of the genital signal in different mice (each curve represents one mouse). For the reliable comparison of signal intensities, the signal intensities were measured from equivalent regions of interest after subtraction of individual backgrounds measured in the right abdominal region.

The main advantage of whole body imaging of luciferase-expressing MHV-68 cycle in living mice is that it reveals novel sites of viral replication. Therefore, we infected 6 weeks-old female BALB/c mice intranasally under general anaesthesia with 10^4^ PFU of luciferase^+^ MHV-68 and tracked infection daily by luciferin injection and charge-coupled-device camera scanning. Representative images are shown in Figure 1A. As previously described [17], [19], [25], we observed signals coming from the nose (d4 p.i.), the thoracic region (d7 p.i.), the neck (d14 p.i.) and the left abdominal region (d14 p.i.). Based on former descriptions [19], [25], [26], we considered the nose signals to come from the nasal turbinates [26]; thoracic signals from the lungs; neck signals from the superficial cervical lymph nodes (SCLNs); and the abdominal signals from the spleen. As previously described [17], [19], [25], the nose and lung signals peaked at 5–7 days after infection and were undetectable after day 14. On the opposite, signal appeared around day 7 in SCLNs and was maximal at day 14, the peak of latency amplification. SCLNs signal then disappeared over the two following weeks. Signals appeared in the spleens around day 10 but were more transient and less often observed than in the SCLNs.

**Figure 2:**
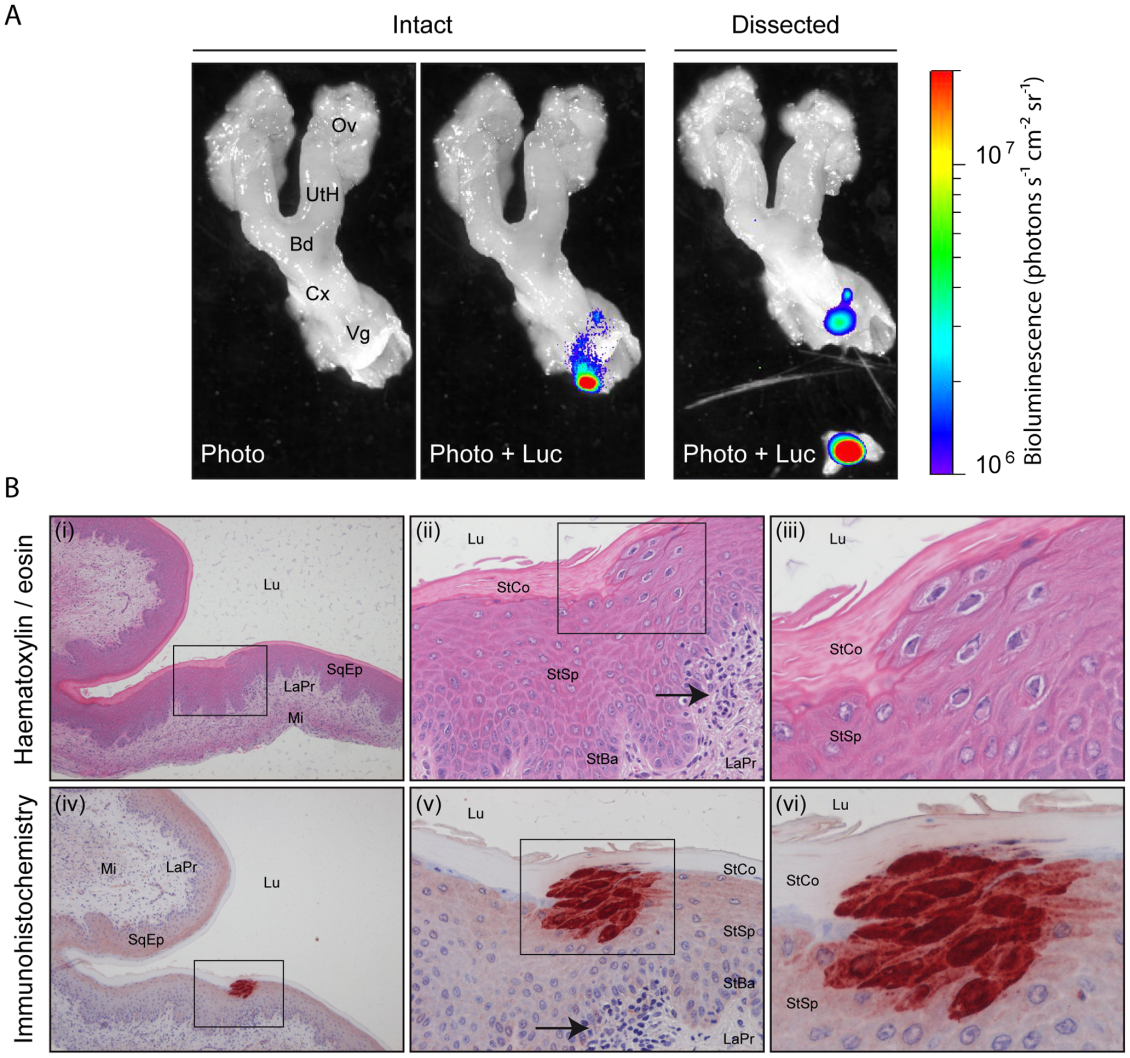
Luciferase signal and MHV-68 antigen detection in isolated genital tract after intranasal infection. **A.** A mouse equivalent to those in Figure 1 was dissected and its genital tract imaged *ex vivo*. The images are representative of data from at least 10 mice, and show either a standard photograph (Photo) or that photograph overlaid with the luciferase signal (Photo+Luc). The region with the highest signal was isolated and processed for histological analysis. The scale bar (photons sec^−1^ cm^−2^ steradian^−1^) shows the color scheme for signal intensity. Ov, ovary; UtH, uterine horn; Bd, body of the uterus; Cx, cervix; Vg, Vagina. **B.** The piece of vagina isolated in A. was fixed in formaldehyde and organ slices were either stained with hematoxylin-eosin (panels i to iii) or processed for immunohistochemistry with anti-MHV-68 rabbit polyserum (panels iv to vi) as described in the Materials and Methods section. Rectangles indicate regions highlighted in the following panels. Arrows indicate focal recruitment of leukocytes at the periphery of MHV-68 antigen detection. Lu, lumen; SqEp, stratified squamous epithelium; LaPr, lamina propria; Mi, Muscularis; StCo, stratum corneum; StSp, stratum spinosum; Stba, stratum basale.

Surprisingly, we randomly observed appearance of luciferase signal in the genital region of infected female mice (Figure 1A–B). This signal appeared after the initial clearance of acute lytic replication in nose and lungs. Moreover, the signal in the genital region was concomitant or appeared after disappearance of the SCLNs and spleen signals (Figure 1A–B and Figure S1). To further investigate MHV-68 replication in the female genital region, we followed it over time among different mice (Figure 1C). Interestingly, ∼80% of the mice displayed luciferase signal in the genital region during this period. This signal was transient (no more than 4 consecutive days) and recurrent. To confirm the sites of infection and to further investigate the origin of the signal, *ex vivo* imaging of individual organs was performed after euthanasia of luciferase^+^ MHV-68 infected mice. This approach revealed that the luciferase signal observed in the genital region was coming from small regions of the vagina (Figure 2A). Fragments of vagina identified as positive for light emission were dissociated from the rest of the organs (Figure 2A) and processed for histological analysis. Immunohistochemical staining for viral antigens identified focal sites of MHV-68 antigen expression in the superior layers of the vaginal epithelium (Figure 2B). This was associated with morphological changes of infected cells (Figure 2B, panel iii) and with the presence of leukocytic infiltrate in the lamina propria (Figure 2B, panels ii and v). These lesions were not observed every time, likely because of their restricted size.

### MHV-68 presence in vagina is associated with release of infectious virions

**Figure 3:**
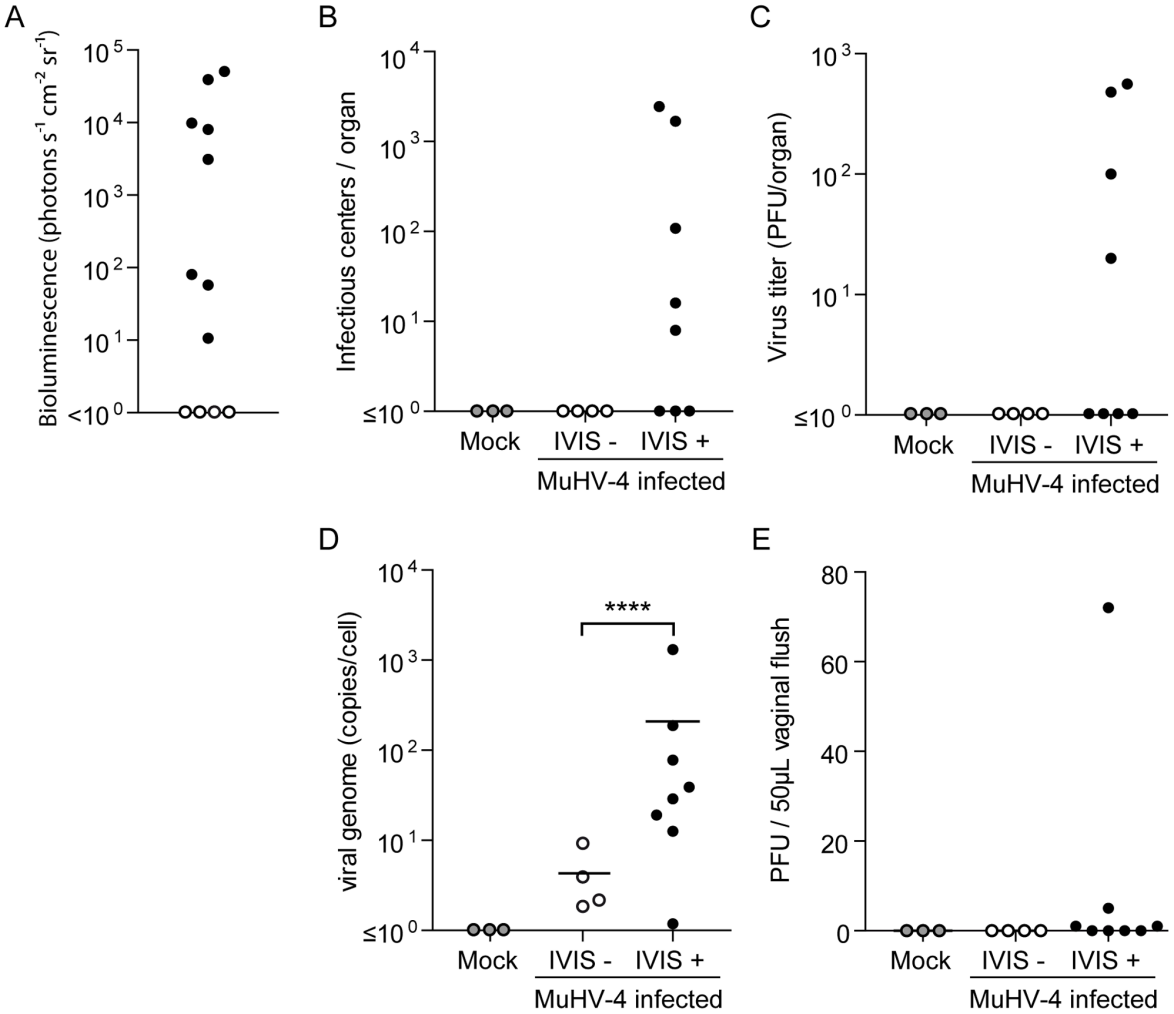
Quantification of MHV-68 infection in female genital tract after intranasal infection. **A.** Female mice were infected intranasally (10^4^ PFU) with WT luciferase^+^ MHV-68 under general anaesthesia. 23 days post-infection, luciferase signal in the genital region was assessed and mice were categorized as IVIS- (white dots) and IVIS+ (black dots). For the reliable comparison of signal intensities, the signal intensities were measured from equivalent regions of interest after subtraction of individual backgrounds measured in the right thoracic region. **B–D.** Individual genital tracts were removed at day 23 p.i. and assayed individually for the presence of MHV-68 by infectious center assay (B), infections virus titration (C) and viral genome quantification (D). Groups were compared by student t-test (****P<0.0001). **E.** Vaginal flush samples collected before euthanasia were tested for the presence of infectious virus. Samples from mock infected mice (grey dots) were used as controls.

In order to further investigate this observation, 12 mice were infected intranasally and light emission from the genital region was measured 23 days p.i. (Figure 3A). This allowed us to categorize mice into two groups: the first in which genital signal was observed was called IVIS+ and the other IVIS-, three uninfected mice were used as mock infected controls. Genital tracts of these mice were isolated as shown in Figure 2A and light emitting regions of the vagina were isolated. Equivalent regions were isolated in mock and IVIS- groups. These different samples were then analyzed by infectious center assays, infectious virus titration and viral genome quantification (Figure 3B–D). These experiments identified the presence of reactivable virus (Figure 3B) and infectious virions (Figure 3C) only in the IVIS+ group. Moreover, there were statistically more copies of MHV-68 genome in the IVIS+ samples than in the IVIS-. Finally, titration of vaginal lavage fluids, collected before euthanasia, revealed the presence of infectious virions in half of the IVIS+ samples (Figure 3E). The latter experiment was repeated on a higher number of mice between days 21 and 30 post-infection (Figure S2A). This revealed that excretion of infectious MHV-68 virions in female genital tracts occurred randomly and was limited in terms of number of PFUs. Finally, in order to establish that nonmanipulated WT MHV-68 parental strain exhibits properties similar to the tagged virus, we have compared viral shedding in the vagina of mice infected either by the WT or by the WT-LUC MHV-68 strains. Our results showed that both viral strains are excreted similarly in the vagina (Figure S2B) for similar latency loads in spleen (Figure S2C). All together, these experiments showed that MHV-68 luciferase signal in female genital tract is associated with the presence of infectious virus in the vaginal epithelium and in the vaginal fluids. Moreover, we observed similar results with the WT parental strain. This could therefore represent a potential portal of transmission of this virus.

### Estrogens indirectly influence MHV-68 excretion in female genital tract

**Figure 4:**
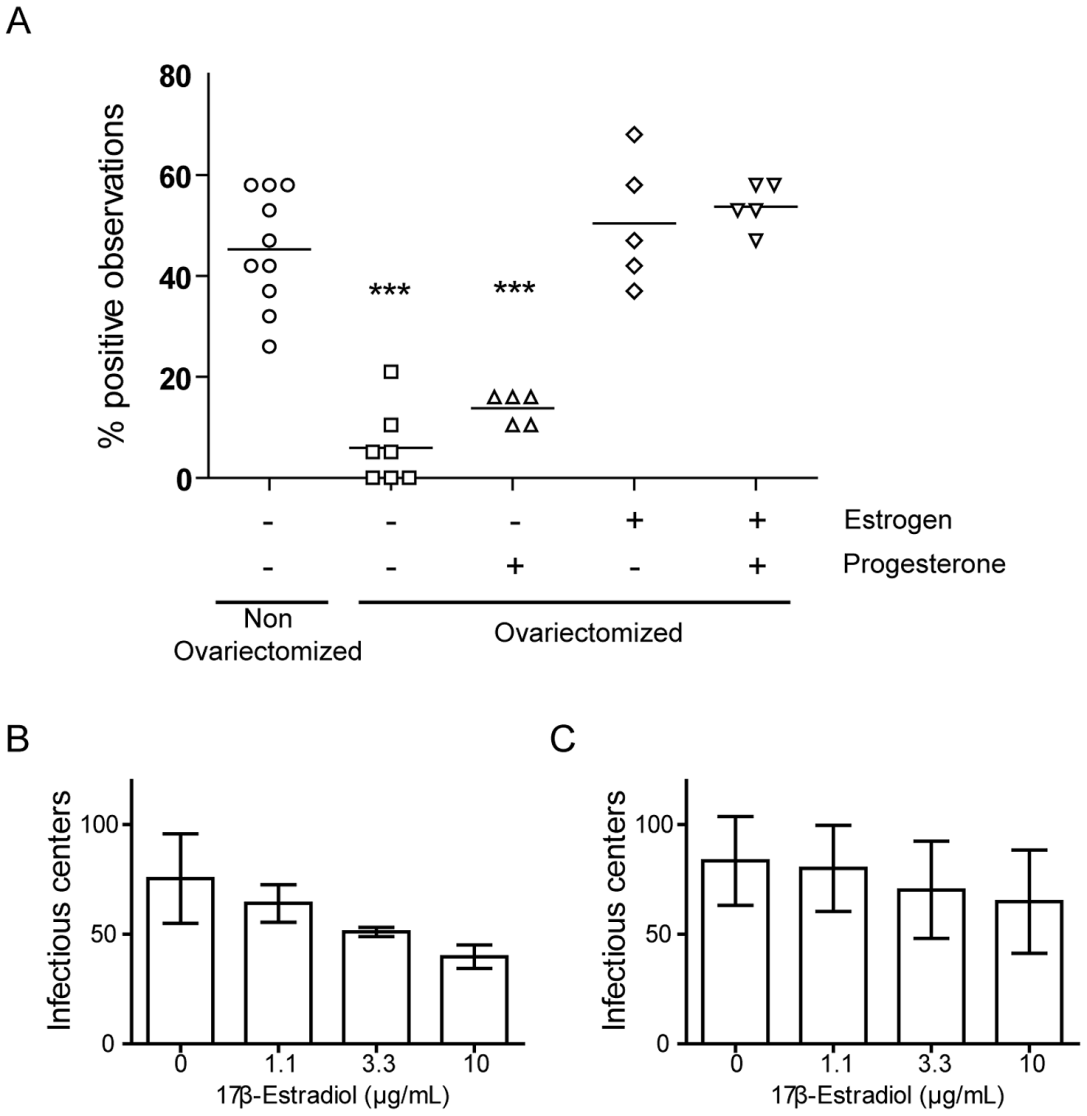
Influence of estrous cycle on genital MHV-68 excretion after intranasal infection. **A.** Control female mice and ovariectomized mice, implanted or not with slow-release hormonal pellets (progesterone and/or estrogen), were infected intranasally (10^4^ PFU) with WT luciferase^+^ MHV-68 under general anaesthesia. Presence of genital signal was monitored between days 14 and 32 post-infection and percentages of positive observations were recorded individually. For the reliable comparison of signal intensities, the signal intensities were measured from equivalent regions of interest after subtraction of individual backgrounds measured daily in the right thoracic region. Each point shows percentage of positive observation for one animal. Groups were compared by ANOVA1 and Bonferroni post-test (***P<0.001). **B–C.** Stimulation of MHV-68 reactivation from persistently infected cells with 17β-Estradiol was tested. MHV-68 persistently infected A20 cells (B) or bulk splenocytes (C), obtained 14 days following MHV-68 intranasal inoculation (10^4^ PFU), were analyzed for the frequency of cells reactivating virus with and without increasing concentrations of 17β-Estradiol as described in the Materials and Methods section. The data presented are the average for triplicate measurements +/− standard error of the mean and were analyzed by 1way ANOVA and Bonferroni post-tests, no statistically significant difference was observed upon treatment.

Random and recurrent observations of MHV-68 associated luciferase signal in female genital tract suggest an association of this phenomenon with the estrous cycle. Indeed, female hormones influence susceptibility, reactivation and transmission of many viruses, including human herpesviruses [27]–[29]. To investigate this possibility, we compared occurrence of MHV-68 associated luciferase signal in genital tract among groups of control and ovariectomized female mice between days 14 and 32 post-infection (Figure 4A and S3). This revealed that ovariectomy greatly diminished observation of MHV-68 associated luciferase expression in the genital tract (Figure 4A and S3) although the normal progression of MHV-68 infection was not affected by the treatments (Figure S4). Indeed, no difference of luciferase signals was observed in lungs at day 7 post-infection between non-ovariectomized and ovariectomized mice (Figure S4A). Similarly, normal lymphoid infection was consistently observed from day 14 to day 21 post-infection (Figure S4B). In order to indentify if it was associated with specific hormonal deprivation, we implanted ovariectomized mice with slow-release progesterone and/or estrogen pellets (Figure 4A and S3). Estrogens alone or in combination with progesterone were sufficient to restore occurrence of genital luciferase signal to levels similar to the ones observed in the non ovariectomized group.

Estrogens could promote genital shedding of MHV-68 by either interacting directly with the infected cell or indirectly, for example by inhibiting the immune response against the virus. To determine whether estrogen treatment can directly trigger MHV-68 reactivation from latently infected cells, we used murine A20 B cells latently infected with MHV-68 (Figure 4B) or explanted splenocytes from MHV-68 infected mice, 14 days p.i. (Figure 4C). These cells were treated with increasing amounts of 17β-Estradiol and MHV-68 reactivation was analyzed by infectious center assays. The results obtained did not show that estrogen stimulation of latently infected cells induces MHV-68 reactivation. The observed effect of estrogens on occurrence of MHV-68 associated luciferase signal (Figure 4A and S3) could therefore be indirect or cell-type specific.

### Genital excretion of MHV-68 is not associated with vertical transmission to the litter or horizontal transmission between female mice

**Figure 5:**
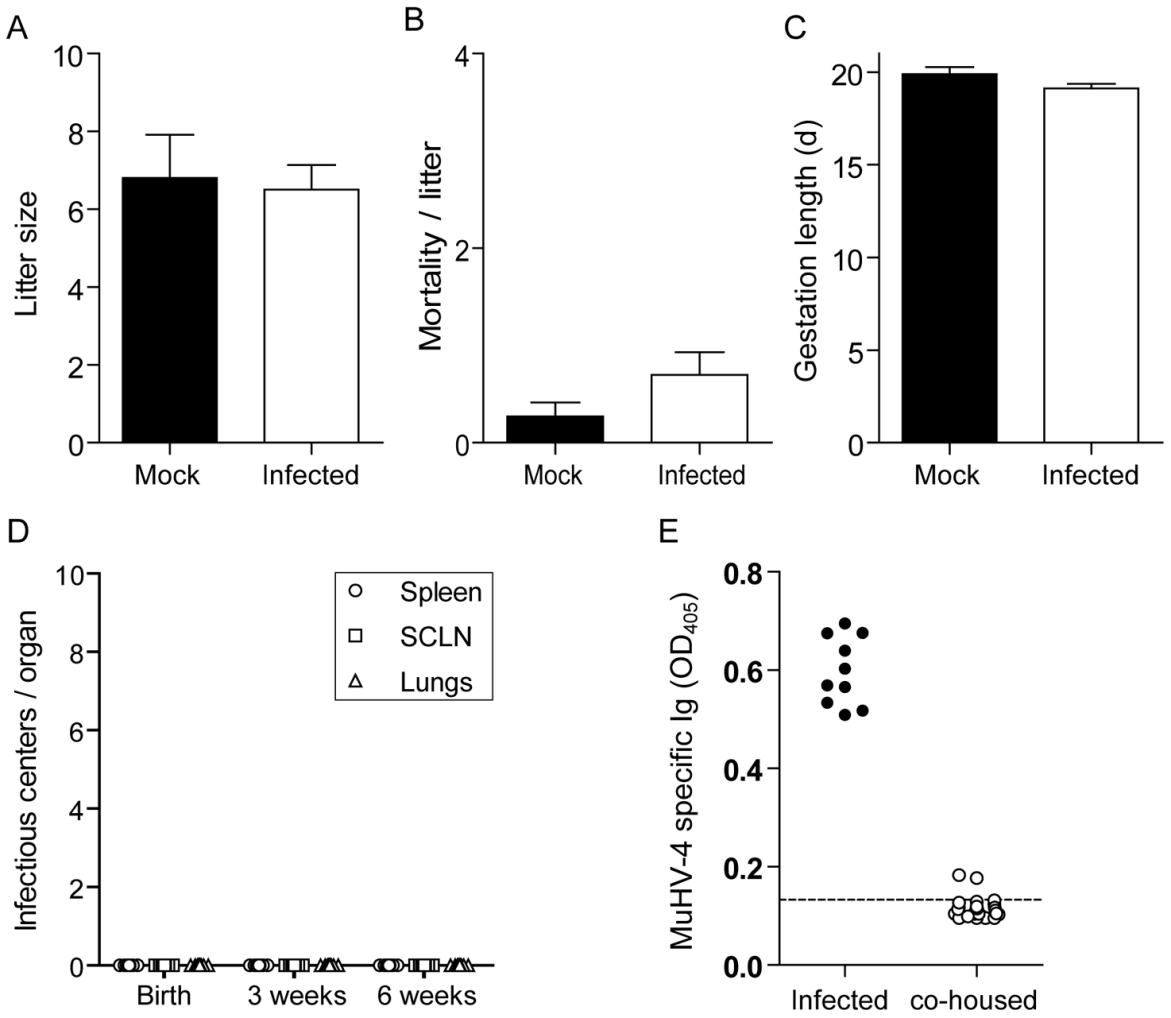
Vertical and non sexual transmissibility of MHV-68 from virus-excreting mice. **A–D.** Female mice were infected intranasally (10^4^ PFU) with WT luciferase^+^ MHV-68 under general anaesthesia, and then injected with luciferin and imaged every day. At the time of the first observation of genital signal, infected females were mated with uninfected males. Mock infected female mice were used as controls. Effect of MHV-68 infection on litter size (A), mortality/litter (B) and gestation length (C) was then monitored. The data presented are the average for 20 (infected) and 11 (Mock) pregnancies +/− standard error of the mean and were analyzed by 1way ANOVA and Bonferroni post-tests, no statistically significant difference was observed. Transmission to the progeny (n≥20 per group) was assessed by infectious center assays performed on isolated organs taken from newborn or at 3 or 6 weeks after birth (C). Data are plotted individually. **E.** Female mice (n = 10) were infected intranasally (10^4^ PFU) with WT luciferase^+^ MHV-68 under general anaesthesia, and then injected with luciferin and imaged every day. At the time of the first observation of genital signal, infected females were co-housed with 3 uninfected females. Potential MHV-68 transmission was monitored 45 days later by detection of anti-MHV-68 specific antibodies. The dashed line indicates the mean value obtained with sera from 3 uninfected mice taken as controls.

The presence of MHV-68 replication in latently infected females could affect gestation. To investigate this hypothesis, luciferase+ MHV-68 infected female mice were mated with uninfected males at the time of the first observation of genital signal. Mock infected female mice were used as controls. Effect of MHV-68 infection on litter size (Figure 5A), mortality/litter (Figure 5B) and gestation length (Figure 5C) was then monitored. We did not observe any effect of MHV-68 infection on any of these parameters (Figure 5A–C). Moreover, we also did not observe transmission to the progeny either at birth or after 3 or 6 weeks (Figure 5D). These results were confirmed by *in vivo* imaging. Briefly, we have imaged latently infected pregnant mothers (n = 30) during gestation (days 18–20 post-mating) and 2 weeks after delivery. Only one pregnant female displayed weak genital signal around delivery (data not shown) whereas all the others (29/30) had no detectable genital signal (Figure S5A and B). 2 weeks after delivery, none of these mice and their offspring displayed any detectable luciferase signal (Figure S5C). Similarly, we also did not observe seroconversion (Figure 5E) or detectable levels of MHV-68 DNA in the spleen of co-housed naïve female mice (data not shown).

### Genital excretion of MHV-68 is associated with sexual transmission

**Figure 6:**
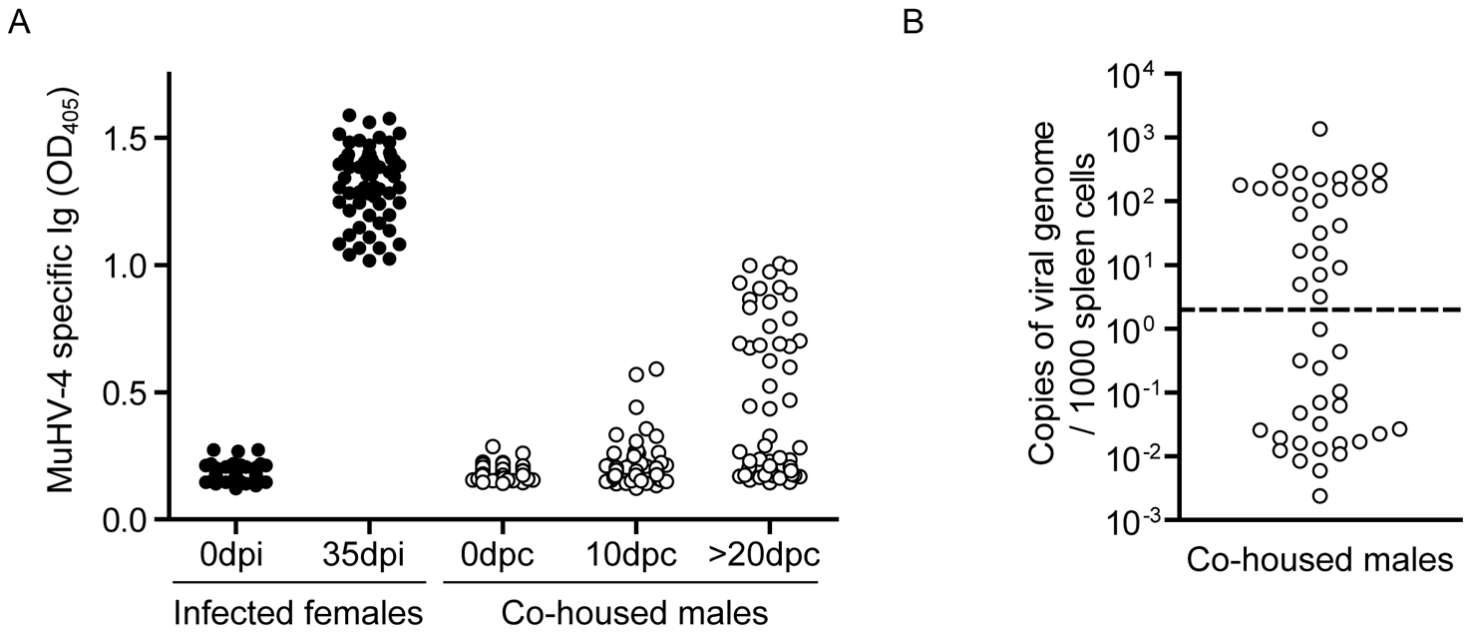
Sexual transmission of MHV-68 from virus-excreting female mice. **A–B.** Female mice were infected intranasally (10^4^ PFU) with WT luciferase^+^ MHV-68 under general anaesthesia, and then injected with luciferin and imaged every day. At the time of the first observation of genital signal, infected females (3 per cages) were mated with uninfected males (3 per cages). MHV-68 infection was monitored at the indicated times by detection of anti-MHV-68 specific antibodies (A) or quantification of viral genomes in male spleens performed after at least 20 days post-contact (B). The dashed line shows the lower limit of the assay sensitivity. Dpi, days post-infection; dpc, days post-contact.

To determine whether the presence of infectious virus in the vaginal epithelium and in the vaginal fluids can result in sexual transmission of MHV-68, we mated luciferase^+^ MHV-68 infected female mice with uninfected males at the time of the first observation of genital signal. We then tested transmission to males by serology at day 10 post-contact and more than 20 days post-contact. Interestingly, we observed seroconversion of 23 individuals among the 60 males that were tested (Figure 6A). As this seroconversion was moderate in comparison to the one observed after intranasal infection (Figure 6A), presence of MHV-68 DNA in spleens was tested. 24 out of these 60 males (among which the 23 that had seroconverted) displayed detectable levels of MHV-68 DNA in the spleen (Figure 6B). All together, these results therefore show that MHV-68 can be transmitted from infected female mice to naïve males.

### Transmission to males is associated with penis infection

**Figure 7:**
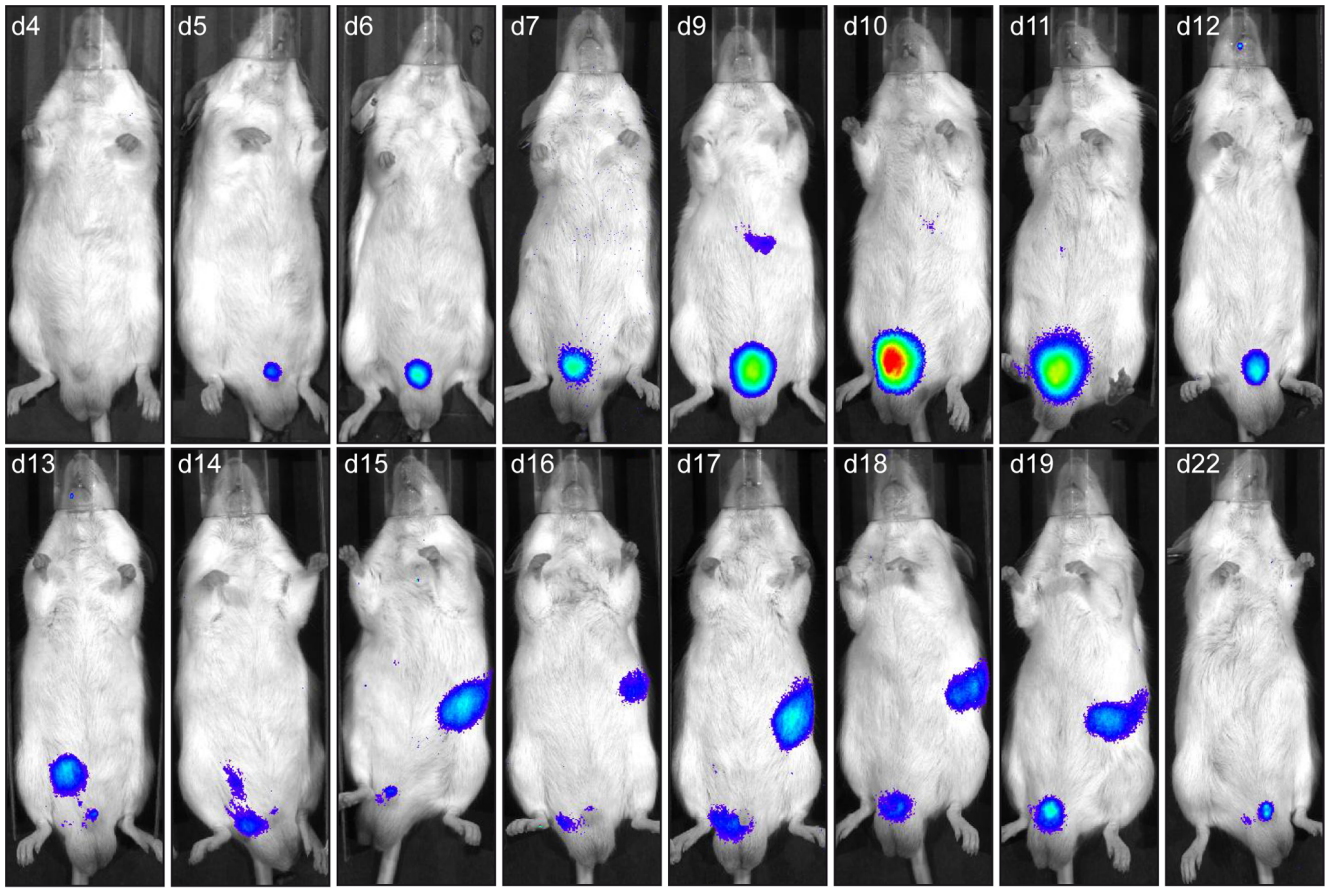
Spatial and temporal progression of MHV-68 infection after sexual transmission to male mice. Female mice were infected intranasally (10^4^ PFU) with WT luciferase^+^ MHV-68 under general anaesthesia, and then injected with luciferin and imaged every day. At the time of the first observation of genital signal, infected females were mated with uninfected males. The males were then injected with luciferin and imaged every day. Images show a representative mouse over time. The day post-contact with the infected female (e.g., d4 is day 4 post-contact) is shown at the top of each image.

**Figure 8:**
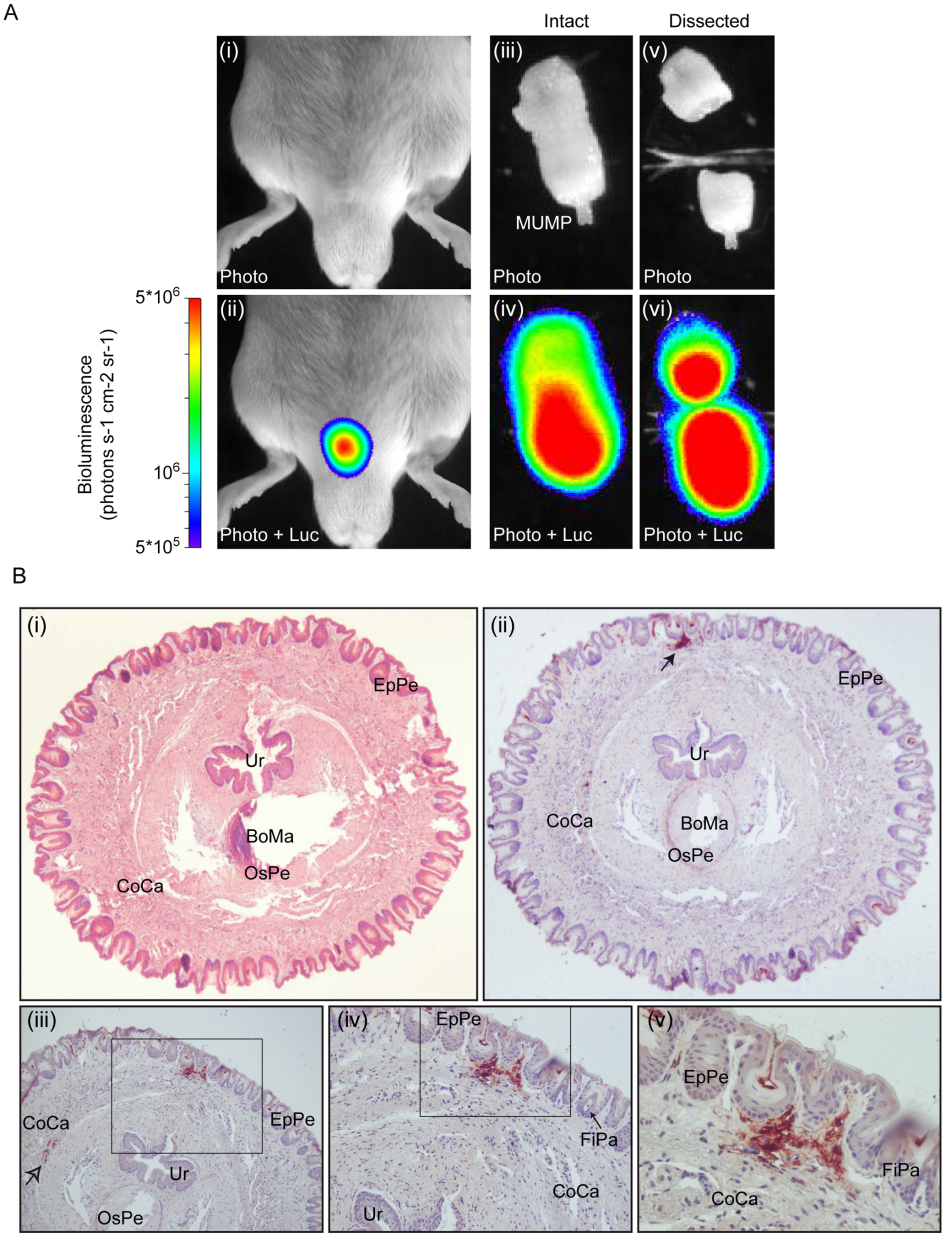
Luciferase signal and MHV-68 antigen detection in isolated male genital tract after sexual transmission. **A.** A mouse equivalent to that in Figure 7 was dissected and its genital tract imaged *ex vivo* at day 10 post-contact with the infected female. The images are representative of data from at least 5 mice, and show either a standard photograph (Photo) or that photograph overlaid with the luciferase signal (Photo+Luc). The region with the highest signal was isolated and processed for histological analysis. The scale bar (photons sec^−1^ cm^−2^ steradian^−1^) shows the color scheme for signal intensity. MUMP, male urogenital mating protuberance. **B.** The piece of penis isolated in A. was fixed in formaldehyde and organ slices were either stained with hematoxylin-eosin (panel i) or processed for immunohistochemistry with anti-MHV-68 rabbit polyserum (panels ii to v) as described in the Materials and Methods section. Rectangles indicate regions highlighted in the following panels. Filled and open arrows indicate detection of MHV-68 antigens in superficial regions of the penis and in deeper region of the Corpus cavernosum, respectively. Ur, urethra; EpPe, Epithelium of the penis; BoMa, Bone marrow; OsPe, Os penis; CoCa, Corpus cavernosum; FiPa, Filiform papilla.

**Figure 9:**
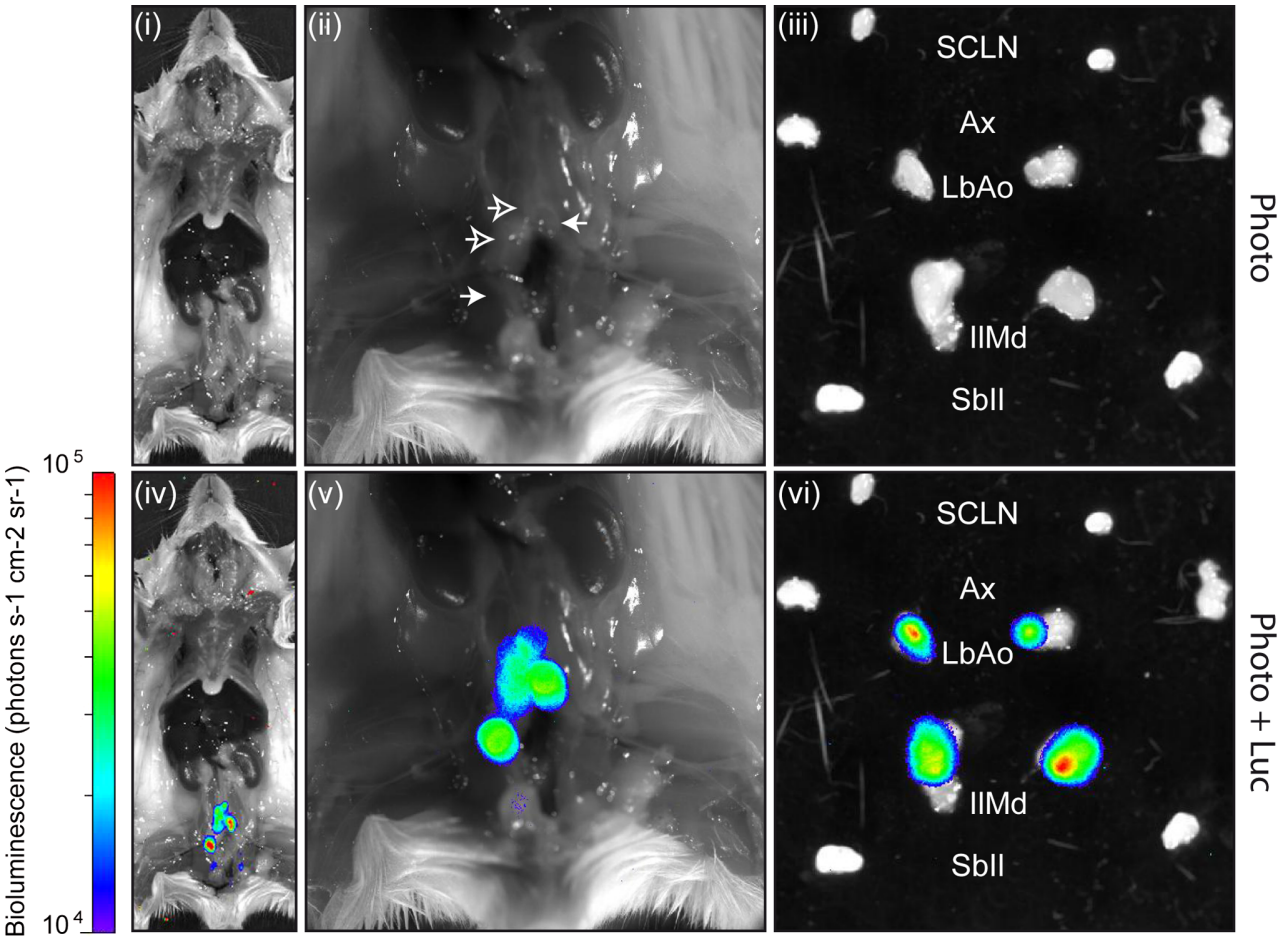
Direct visualization of lymph node colonization after sexual transmission of MHV-68 infection. A mouse equivalent to those in Figure 8 and 9 was dissected 2 weeks post-contact with the infected female. Representative images show either a standard photograph (Photo) or that photograph overlaid with the luciferase signal (Photo+Luc). The entire body (panels i and iv) and the pelvis region (panels ii and v) are shown after displacement of the genital tract. Panels iii and vi show isolated lymph nodes. The scale bar (photons sec^−1^ cm^−2^ steradian^−1^) shows the color scheme for signal intensity. SCLN, superficial cervical lymph nodes; Ax, axillary lymph nodes; LbAo, lumbar aortic lymph nodes; IlMd, medial iliac lymph nodes; SbIl, subiliac lymph nodes.

To determine the route of MHV-68 transmission to naïve males, we repeated the previous experiment and tracked MHV-68 infection of males daily by luciferin injection and charge-coupled-device camera scanning (Figure 7). We observed that light emission appeared in the genital region around 4 days post-contact. This signal peaked around 10 days post-contact but was maintained for at least 3 weeks. To confirm the site of infection and to further investigate the origin of the signal, *ex vivo* imaging of individual organs was performed after euthanasia of luciferase^+^ MHV-68 infected males at different time points. This approach revealed that the luciferase signal observed in the genital region was coming from small regions of the penis (Figure 8A). Fragments of the penis identified as positive for light emission were dissociated from the rest of the organ (Figure 8A) and processed for histological analysis. Immunohistochemical staining for viral antigens identified focal sites of MHV-68 antigen expression in the superior layers of the penis epithelium and of the corpus cavernosum (Figure 8B). Viral antigens were also detected in deeper regions of the corpus cavernosum (Figure 8B, panel iii). Penis infection was associated with propagation of the infection to draining lymph nodes. *Ex vivo* imaging revealed that they were mainly lumbar aortic medial iliac lymph nodes (Figure 9). Light emitted by these lymph nodes had already been observed during imaging of living animals (Figure 7, days 13 to 17). Finally, colonization of the spleen was observed (Figure 7, days 15 to 19) as already showed by viral genome detection (Figure 6B). Interestingly, genital signal in males was never observed after intra-nasal infection (Figure S6).

### Virus transmission from genitally infected males to naïve females

We studied female to male transmission to validate the physiological significance of virus shedding from the female genital tract. We saw no virus shedding from the male genital tract after intranasal infection (Figure S6), so the requirements and routes of male to female transmission are less clear. To explore it we relied on the acute genital signal of infected males. Thus, we infected 30 female mice intranasally (10^4^ PFU) with luciferase+ MHV-68, then imaged them each day by luciferin injection and CCD camera scanning. When genital signal was observed, infected females (3 per cage) were mixed with uninfected males (3 per cage). Male infection was then monitored by luciferase imaging and by serology at 10 and 20 days post-contact. We observed genital luciferase signal and positive serology in 13/30 males. These results are incorporated into Figure 6A. Luciferase+ males were then mixed with uninfected females (3 per male). We did not observe luciferase signal in these females, but one seroconverted (Figure S9) and infection was confirmed by Q-PCR of viral DNA from the spleen (10.4 viral genome copies/1000 spleen cells). Low dose intranasal infection (10 p.f.u.) often leads to seroconversion without detectable nasal luciferase signal (data not shown). Thus this was a bona fide infection, but by an undetermined route.

## Discussion

Transmission in host population is the main motor of viral evolution [30]–[32]. Herpesviruses have co-evolved with their host for millions of years and have therefore developed sophisticated mechanisms to persist and transmit in presence of protective immune response [33], [34]. This is particularly the case for gammaherpesviruses [20], [35], [36]. Until now, most of the immune evasion strategies of gammaherpesviruses have been studied *in vitro* or in animal models [20], [35], [36]. However, none has been investigated in the light of transmission mainly due to the lack of experimental transmission model. In this study, using *in vivo* imaging, we observed that MHV-68 is genitally excreted after latency establishment in intranasally infected female mice (Figures 1–3, S1 and S2). This allowed us to observe, for the first time, experimental transmission to naïve males after sexual contact (Figures 6–9).

The observation of vaginal shedding of MHV-68 is somewhat surprising as numerous people have been working on this model around the world for a long time without reporting such observations. However, several points can be mentioned. First, we have used a method of *in vivo* imaging that was recently developed and which is particularly sensitive, allowing the detection of low levels of replicative virus [17], [37]. Secondly, we have followed the infections daily and during a long period, generally between 14 and 32 days post infection. To our knowledge, such following of the infectious process has never been reported. Interestingly, the two previous studies using *in vivo* luciferase imaging of MHV-68 cycle suggested potential genital infection. Milho *et al.* showed that the female genital tract is a site of virus replication after intraperitoneal infection [19] and one of the mice used by Hwang *et al.* displayed light emission in the genital region after intranasal infection and latency establishment (Hwang et al., Figure 2A, day 18 p.i. [25]). The high frequency of genital signal observation in our study (∼80% of the infected mice) could reflect particular experimental conditions. For example, a potential co-infection with another pathogen could favour MHV-68 genital excretion. Such synergic relation has been demonstrated for others herpesviruses, notably HSV-2 and human cytomegalovirus, with HIV-1 [38]–[41]. If such a pre-existent infection exists, the causal agent remains to be identified. Another explanation could be related to our housing facility which homes both females and males mice. This can be an important element as male pheromones can modulate estrous cycle in mice [42].

The observation of genital signal in females was dependent on the estrous cycle as ovariectomy nearly abolished the phenomenon and as estrogens supplementation restored it (Figure 4). Steroid hormones influence susceptibility, replication as well as transmission of many viruses, including herpesviruses [43], [44]. Numerous studies have illustrated the influence of female sex hormones on both susceptibility and immune responses to sexually transmitted pathogens [43]–[45]. Thus, estrogen and progesterone influence the susceptibility to genital herpes infection [43], [44]. However, in those cases the presence of progesterone increased susceptibility to HSV-2 [46], whereas the presence of estrogen prevented or decreased the risk of HSV infection in the female genital tract [47]–[49]. While these hormones can directly influence the sensitivity of the cells of the genital tract, they could also attenuate or modulate the innate [50], [51] and/or the adaptive [44] immune response against the virus. For example, the abundance of antigen presenting cells, T cells and B cells has been shown to vary in uterus and vagina with the estrous cycle [52]–[54]. Replication and shedding of MHV-68 could therefore be a consequence of impaired immune surveillance. On another hand, as some of these immune cells harbour MHV-68 latent infection [21], [36], the transient observation of genital signal in females could reflect the variation of abundance of some particular cell types over time. In the future, these point will have to be tackled. Thus, the origin of vaginally excreted virions could be addressed by using cell-type specific Cre/Lox genetic labelling of MHV-68 to track the route of viral excretion *in vivo* as it has recently been done to explore the host colonization pathway [26]. Interestingly, the fact that the MHV-68-associated luciferase genital signal lasted for at most 3–4 days (Figure 1C) could be linked to the cyclic remodelling of the epithelium observed during the estrous cycle. Indeed, infected cells are located in the superior layers of the vaginal epithelium (Figure 2B) and could therefore be removed at each cycle.

Besides these indirect roles, steroid hormones have also been shown to directly induce herpesvirus reactivation from latency. Thus, 17β-estradiol promotes HSV-1 reactivation in latently infected neurons [29]. Similarly, several studies have shown that dexamethasone, a synthetic corticosteroid, induces Bovine Herpesvirus-1 reactivation from latency either *in vitro* or *in vivo* in calves [55] and rabbits [56]. This has been associated with the induction of cellular transcription factors and/or signalling pathways that stimulate viral lytic genes expression and subsequent reactivation [57]–[60]. In the present study, we did not observe any direct effect of estrogens on latently infected B-cells either *in vitro* or *ex vivo* (Figure 4 B and C). However, we have no evidence that these cells mimic what happens *in vivo* in the infected female genital tract. Further experiments are therefore required to identify the mechanism involved in estrogen-induced MHV-68 vaginal shedding.

In males, initial infection was localized in the superior layers of the penis epithelium and of the corpus cavernosum (Figure 8B). Infection then spreads to draining lymph nodes and spleen (Figure 7 and 9). Again, cell-type specific Cre/Lox genetic labelling of MHV-68 [26] will be helpful to track the route of viral infection after sexual transmission. As infectious virions were rarely detected in vaginal lavages although MHV-68 induced luciferase signal was frequent, we hypothesize that close contacts between genital organs of males and females are necessary to transmit infection. Indeed, the penis of the male mice is recovered of spines called filiform papilla. These structures could therefore induce abrasion of the vaginal epithelium and promote virus transmission. Interestingly, cells that were initially infected on penis were located around these filiform papilla (Figure 8B). Infection persisted at this site for at least three weeks (Figure 7). The importance of this observation for MHV-68 epidemiology will therefore have to be tested. For example, it has recently been shown that male circumcision significantly reduces the incidence of HSV-2 and HIV-1 infection and the prevalence of HPV infection [61], [62]. Our results suggest that it could also be the case for some gammaherpesviruses.

Until now, we did not manage to establish experimental conditions to repeatedly transmit the virus from genitally infected males to naïve females. Human herpesvirus transmission generally occurs at a low rate even between close contacts [63]. However, our results (Figure S7) can only be considered suggestive of male to female MuHV-4 transmission. Important aspects of mouse behaviour such as scent marking may not be properly reproduced with conventional housing. We conclude that under the experimental conditions used, male to female transmission is possible but inefficient, certainly much less so that female to male transmission. The normal mode of male to female MuHV-4 transmission remains to be determined.

Sexual transmission constitutes an easy way of spread for a virus in natural populations of wild animals. This is particularly the case for rodents. Indeed, rodents live generally in small groups spread on a relatively large territory. Sexual contact could therefore be a relatively efficient route of transmission. Interestingly, Telfer *et al.* showed that gammaherpesvirus (identified serologically as MuHV-4, though likely Wood Mouse Herpesvirus) infection in wood mice was more prevalent in heaviest, sexually active, males than in any other category of animal [64]. The fact that the viral shedding in the female genital tract is linked to sexual cycle and more precisely to the period of estrus (high rates of estrogens) would be very beneficial for transmission as re-excretion would occur during the periods of female receptivity for mating. Sexual transmission has also been proposed for EBV and KSHV [9], [65], [66] but is mainly important for HSV-1 and -2 [67]. The observation of MHV-68 sexual transmission from infected females to naïve males could therefore be particularly interesting in the general context of herpesvirus transmission.

Shedding of MHV-68 in the female genital tract could also have an effect on progeny. However, in contrast to what was reported by Stiglincova *et al.* [68], we did not observe premature termination of pregnancy, reduced number of newborns, vertical transmission or transmission through milk of MHV-68 in mice (Figure 5). We have no explanation for this discrepancy. However, mother to child transmission of human gammaherpesviruses, both transplacental or perinatal, seems also to be very limited [9], [69].

The identification of a route of transmission for MHV-68 in mice opens new fundamental research perspectives. Thus, it will allow testing the importance of various immune evasion strategies, such as those based on the gp150 glycoprotein [70], [71] in the light of transmission. It will also be interesting to test if transmission requires latency establishment and reactivation of the virus or, conversely, if it is enhanced by immunosuppression through the use of drugs like Cyclosporine A [25] or of depletion of specific cell types such as CD8 [72]. Finally, it will be possible to test antiviral and/or vaccinal strategies in the context of infection epidemiology.

Altogether, in this study we identified for the first time a genital excretion site of MHV-68 after latency establishment in intranasally infected female mice. This has allowed us to observe sexual transmission of the virus from infected females to naïve males. These results open new perspectives for the study of gammaherpesvirus in particular but also for the study of sexually transmitted infections in general.

## Materials and Methods

### Ethics statement

The experiments, maintenance and care of mice complied with the guidelines of the European Convention for the Protection of Vertebrate Animals used for Experimental and other Scientific Purposes (CETS n° 123). The protocol was approved by the Committee on the Ethics of Animal Experiments of the University of Liège, Belgium (Permit Number: 1051). All efforts were made to minimize suffering.

### Animals

Females and males BALB/c mice were purchased from Charles River Laboratories. All the animals were housed in the University of Liège, Department of infectious diseases. The animals were infected with MHV-68 when 6–12 weeks old. Intranasal infections with anaesthesia were in 30 µl aliquots. For luciferase imaging, animals were anaesthetized with isoflurane, injected intraperitoneally with luciferin (150 mg/kg), then scanned with an IVIS Spectrum (Caliper Life Sciences). Animals were routinely imaged after 10 min. For quantitative comparisons, we used Living Image software (Caliper Life Sciences) to obtain the maximum radiance (photons per s per cm^2^ per steradian, i.e. photons s^−1^ cm^−2^ sr^−1^) over each region of interest.

### Cells and virus

We used the MHV-68 strain of MuHV-4 [16] and a MHV-68 strain expressing luciferase under control of the M3 promoter that was described previously (hereafter called WT-LUC strain) [19]. Briefly, a luciferase expression cassette was inserted between the polyadenylation signals of ORFs57 and 58. This viral strain did not show any growth deficit either *in vitro* or *in vivo* [19]. Viruses used in this study were propagated on BHK-21cells cultured in Dulbecco's modified Eagle's medium (Invitrogen) and supplemented with 2 mM glutamine, 100 U penicillin ml^−1^, 100 mg streptomycin ml^−1^ and 10% fetal calf serum. Virions were concentrated as described previously [70].

### Viral infectivity assays

Virus stocks were titrated by plaque assay on BHK-21 cells [73]. Cell monolayers were incubated with virus (2 h, 37°C), overlaid with 0.3% carboxymethylcellulose (CMC, medium viscosity, Sigma), and 4 days later fixed and stained for plaque counting [74]. Infectious virus in organs was measured by homogenizing them after freezing (−80°C) in 6 ml complete medium prior to plaque assay.

### Virus detection by infectious centre assay

Virus detection in genital organs cell suspension was assayed by infectious centre assay (ICA) as follows. 5.10^5^ BHK-21 cells grown in 6 well cluster dishes (Becton Dickinson) were co-cultured for 5 days at 37°C with ex vivo cell suspension in MEM containing 10% FCS, 2% PS, 0.3% CMC and 5.10^−5^ M of β-mercaptoethanol (Merck). Cells were then fixed and stained for plaque counting.

### Viral genome quantification

Viral genome loads were measured by real-time PCR [17]. DNA from organs (100 ng) was used to amplify MHV-68 genomic co-ordinates 4166–4252 (iCycler, Biorad) (gene M2, forward primer 5′- GTCAGTCGAGCCAGAGTCCAACA-3′, reverse primer 5′-ATCTATGAAACTGCTAACAGTGAAC-3′). The PCR products were quantified by hybridization with a TaqMan probe (genomic co-ordinates 4218–4189, 5′ 6-FAM-TCCAGCCAATCTCTACGAGGTCCTTAATGA-BHQ1 3′) and converted to genome copies by comparison with a standard curve of cloned plasmid template serially diluted in control spleen DNA and amplified in parallel. Cellular DNA was quantified in parallel by amplifying part of the interstitial retinoid binding protein (IRBP) gene (forward primer 5′-ATCCCTATGTCATCTCCTACYTG-3′, reverse primer 5′-CCRCTGCCTTCCCATGTYTG-3′). The PCR products were quantified with Sybr green (Invitrogen), the copy number was calculated by comparison with standard curves of cloned mouse IRBP template amplified in parallel. Amplified products were distinguished from paired primers by melting curve analysis and the correct sizes of the amplified products confirmed by electrophoresis and staining with ethidium bromide.

### Detection of infectious virus in vaginal fluids

Vaginal lavage fluids were obtained by gentle flushing of the mouse vagina with 100 µl of sterile PBS. Lavage fluids were then centrifuged and the supernatant was titrated as described above.

### Ovariectomy and hormonal supplementation

Ovariectomy were performed at 3 weeks of age under isoflurane anaesthesia. Hormonal treatment was started 3 weeks after ovariectomy. 60 days slow-release pellets (Innovative Research of America, Sarasota, FL, USA) containing 0.05 mg 17β-estradiol (SE-121), or 25 mg progesterone (SP-131) per pellet were implanted subcutaneously, giving a release of ∼0.8 µg 17β-estradiol or ∼400 µg progesterone per 24 hours.

### *In vitro* and *ex vivo* hormonal stimulation

17-β-estradiol (Sigma) stock solution was prepared in DMSO (1 mg/ml). For *in vitro* stimulation, A20-Syndecan-1 cells [75] were persistently infected with a MHV-68 strain expressing eGFP under an EF1a promoter, between the 3′ ends of ORFs 57 and 58 (Dr P.G. Stevenson, unpublished data). For *ex vivo* stimulation, spleen of WT MHV-68 intranasally infected mice were harvested 14 days post-infection, cells were dissociated and erythrocytes were lysed by using red blood cells lysis buffer. Cells were cultivated in RPMI medium without phenol red, to avoid the presence of steroids, supplemented with 2 mM glutamine, 100 U penicillin ml^−1^, 100 mg streptomycin ml^−1^, 5*10^−5^ M of β-mercaptoethanol (Merck) and 10% Charcoal Stripped Fetal Bovine Serum (CSFBS, Sigma). Stimulation of virus reactivation by 17-β-estradiol was performed as follows. Briefly, 3*10^5^ BHK-21 cells grown in 6 well cluster dishes were co-cultured for 5 days at 37°C with 5*10^3^ MHV-68 infected A20 cells or 5*10^5^ infected spleen cells in RPMI containing 10% CSFBS, 2% PS, 0.3% CMC, 5.10^−5^ M of β-mercaptoethanol (Merck) and complemented with increasing doses of 17-β-estradiol. After 5 days, cells were fixed and stained for plaque counting.

### Organ histology and immunohistochemistry

Portions of genital organs were fixed in buffered formol saline, processed routinely to 5-mm paraffin wax-embedded sections, stained with hematoxylin and eosin, and examined by light microscopy. Immunohistochemistry was performed using EnVision Detection Systems (DAKO) with anti-MHV-68 rabbit hyperimmune serum against MHV-68 as primary antibody [17].

### Quantification of anti-MHV-68 specific antibodies by ELISA

Nunc Maxisorp ELISA plates (Nalgene Nunc) were coated for 18 h at 37°C with 0.1% Triton X-100-disrupted MHV-68 virions (2.10^6^ PFU/well), blocked in PBS/0.1% Tween-20/3% BSA, and incubated with mouse sera (diluted 1/200 in PBS/0.1% Tween-20/3% BSA). Bound antibodies were detected with Alkaline Phosphatase conjugated goat anti-mouse Ig polyclonal antibody (Sigma). Washing were performed with PBS/0.1% Tween-20/3% BSA. p-Nitrophenylphosphate (Sigma) was used as substrate and absorbance was read at 405 nm using a Benchmark ELISA plate reader (Thermo).

## Supporting Information

Figure S1 ***In vivo*** **infection by luciferase-expressing MHV-68.** Female mice were infected intranasally (10^4^ PFU) with WT luciferase^+^ MHV-68 under general anaesthesia, and then injected with luciferin and imaged every days. Images show representative mice around 2 weeks p.i. The scale bar (photons sec^−1^ cm^−2^ steradian^−1^) shows the color scheme for signal intensity.
(TIF)

Figure S2 **Quantification of infectious MHV-68 virions in vaginal flushes after intranasal infection.** **A.** Female mice were infected intranasally (10^4^ PFU) with WT luciferase^+^ MHV-68 under general anaesthesia. Individual vaginal flush samples (at least 10 per time point) were collected between day 21 and 30 p.i. and were tested for the presence of infectious virions as described in the Material and Methods. **B–C.** Female mice (n = 10) were infected intranasally (10^4^ PFU) with either the WT luciferase^+^ or the parental WT strain of MHV-68 under general anaesthesia. Individual vaginal flush samples were collected between day 14 and 30 p.i. and were tested for the presence of infectious virions as described in the Material and Methods (**B**). Spleens from these mice were analysed for viral genomes by real-time PCR. Each bar shows the mean viral genome copy numbers per host genome +/− standard deviation (SD) for each group of 10. No statistical difference was observed between groups (Student t-test).
(TIF)

Figure S3 **Influence of estrous cycle on genital MHV-68 excretion after intranasal infection.** Control female mice and ovariectomized mice, implanted or not with slow-release hormonal pellets (progesterone and/or estrogen), were infected intranasally (10^4^ PFU) with WT luciferase^+^ MHV-68 under general anaesthesia. Individual genital signals were monitored between days 14 and 32 post-infection. For the reliable comparison of signal intensities, the signal intensities were measured from equivalent regions of interest after subtraction of individual backgrounds measured daily in the right thoracic region. Each point shows one measurement. 5 individual mice per group are shown.
(TIF)

Figure S4 **Influence of estrous cycle on lung and SCLN luciferase signals after intranasal infection.** Control female mice and ovariectomized mice, implanted or not with slow-release hormonal pellets (progesterone and/or estrogen), were infected intranasally (10^4^ PFU) with WT luciferase^+^ MHV-68 under general anaesthesia. **A.** Presence of lung signals was monitored at day 7 post-infection. The data presented are the average for triplicate measurements +/− standard error of the mean and were analyzed by 1way ANOVA and Bonferroni post-tests, no statistically significant difference was observed upon treatment. **B.** Lymphoid infection was monitored from day 14 to day 21 post-infection. The data presented are the average for triplicate measurements +/− standard error of the mean and were analyzed by 1way ANOVA and Bonferroni post-tests, no statistically significant difference was observed upon treatment.
(TIF)

Figure S5 **Luciferase signal in pregnant mice and their offspring.** Female mice were infected intranasally (10^4^ PFU) with WT luciferase^+^ MHV-68 under general anaesthesia, and then injected with luciferin and imaged every day. At the time of the first observation of genital signal, infected females were mated with uninfected males. **A.** Pregnant females were then injected with luciferin and imaged around day 20 post-mating. Images show 5 representative mice. **B.** The same mice were imaged similarly the day after delivery. Images show 5 representative mice. **C.** These mice and their litter were finally injected with luciferin and imaged 2 weeks post-delivery. Images show 3 representative mice with three of their pups. The scale bars (photons sec^−1^ cm^−2^ steradian^−1^) show the color scheme for signal intensity.
(TIF)

Figure S6 ***In vivo*** **intranasal infection of males mice by luciferase-expressing MHV-68.** 8 weeks-old male BALB/c mice (n = 10) were infected intranasally (10^4^ PFU) with WT luciferase^+^ MHV-68 under general anaesthesia, and then injected with luciferin before *in vivo* imaging. Images show a representative mouse at days 0, 7, 14, 16, 18, 20 and 22 p.i.. The scale bar (photons sec^−1^ cm^−2^ steradian^−1^) shows the color scheme for signal intensity.
(TIF)

Figure S7 **MHV-68 transmission from genitally infected males to naïve females.** Genitally infected males (n = 13) (infected after contact with infected females excreting the virus in the genital tract) were mixed with uninfected females (at least 3 per male). MHV-68 infection of females was monitored 18 days post-contact by detection of anti-MHV-68 specific antibodies as described in the Material and Methods.
(TIF)
