# Supplementary figures and images for: Illumination of Murine Gammaherpesvirus-68 Cycle Reveals a Sexual Transmission Route from Females to Males in Laboratory Mice
Source: PLoS Pathog. 2013 Apr 4;9(4):e1003292. doi: 10.1371/journal.ppat.1003292 (PMC3616973; doi:10.1371/journal.ppat.1003292)

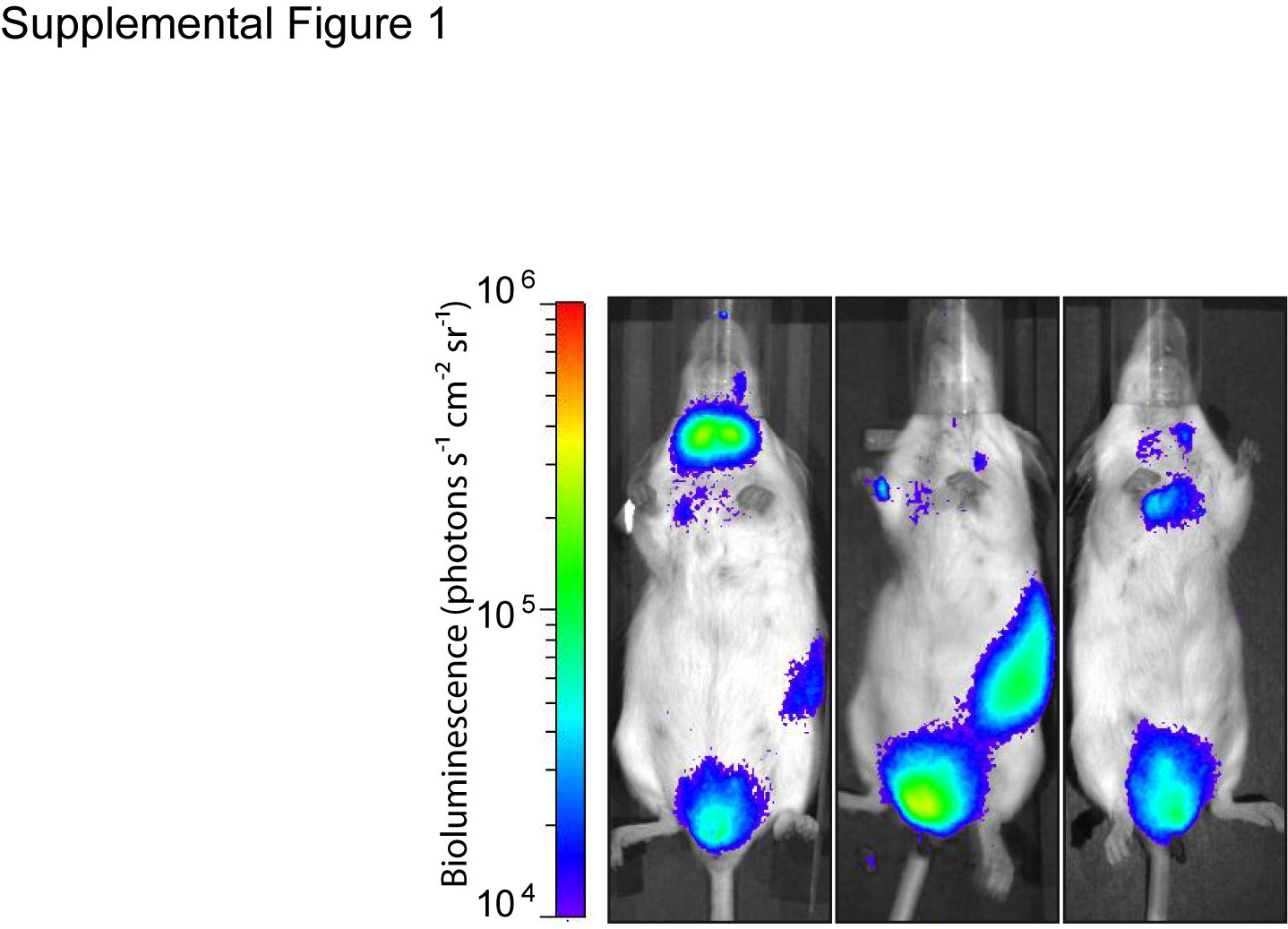

Supplement: Figure S1 — In vivo infection by luciferase-expressing MHV-68. Female mice were infected intranasally (104 PFU) with WT luciferase+ MHV-68 under general anaesthesia, and then injected with luciferin and imaged every days. Images show representative mice around 2 weeks p.i. The scale bar (photons sec−1 cm−2 steradian−1) shows the color scheme for signal intensity. (TIF) [file ppat.1003292.s001.tif]

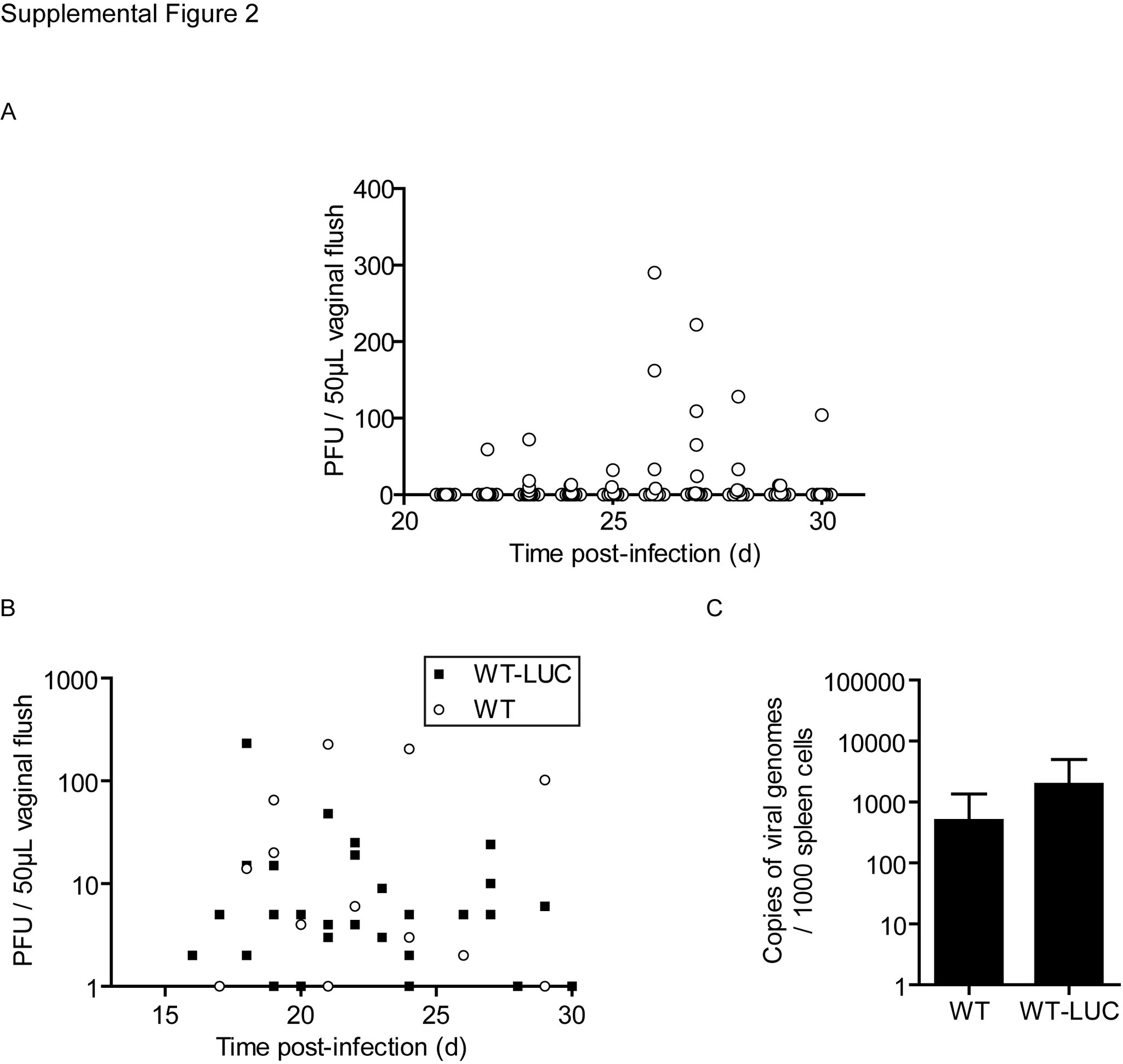

Supplement: Figure S2 — Quantification of infectious MHV-68 virions in vaginal flushes after intranasal infection. A. Female mice were infected intranasally (104 PFU) with WT luciferase+ MHV-68 under general anaesthesia. Individual vaginal flush samples (at least 10 per time point) were collected between day 21 and 30 p.i. and were tested for the presence of infectious virions as described in the Material and Methods. B–C. Female mice (n = 10) were infected intranasally (104 PFU) with either the WT luciferase+ or the parental WT strain of MHV-68 under general anaesthesia. Individual vaginal flush samples were collected between day 14 and 30 p.i. and were tested for the presence of infectious virions as described in the Material and Methods (B). Spleens from these mice were analysed for viral genomes by real-time PCR. Each bar shows the mean viral genome copy numbers per host genome +/− standard deviation (SD) for each group of 10. No statistical difference was observed between groups (Student t-test). (TIF) [file ppat.1003292.s002.tif]

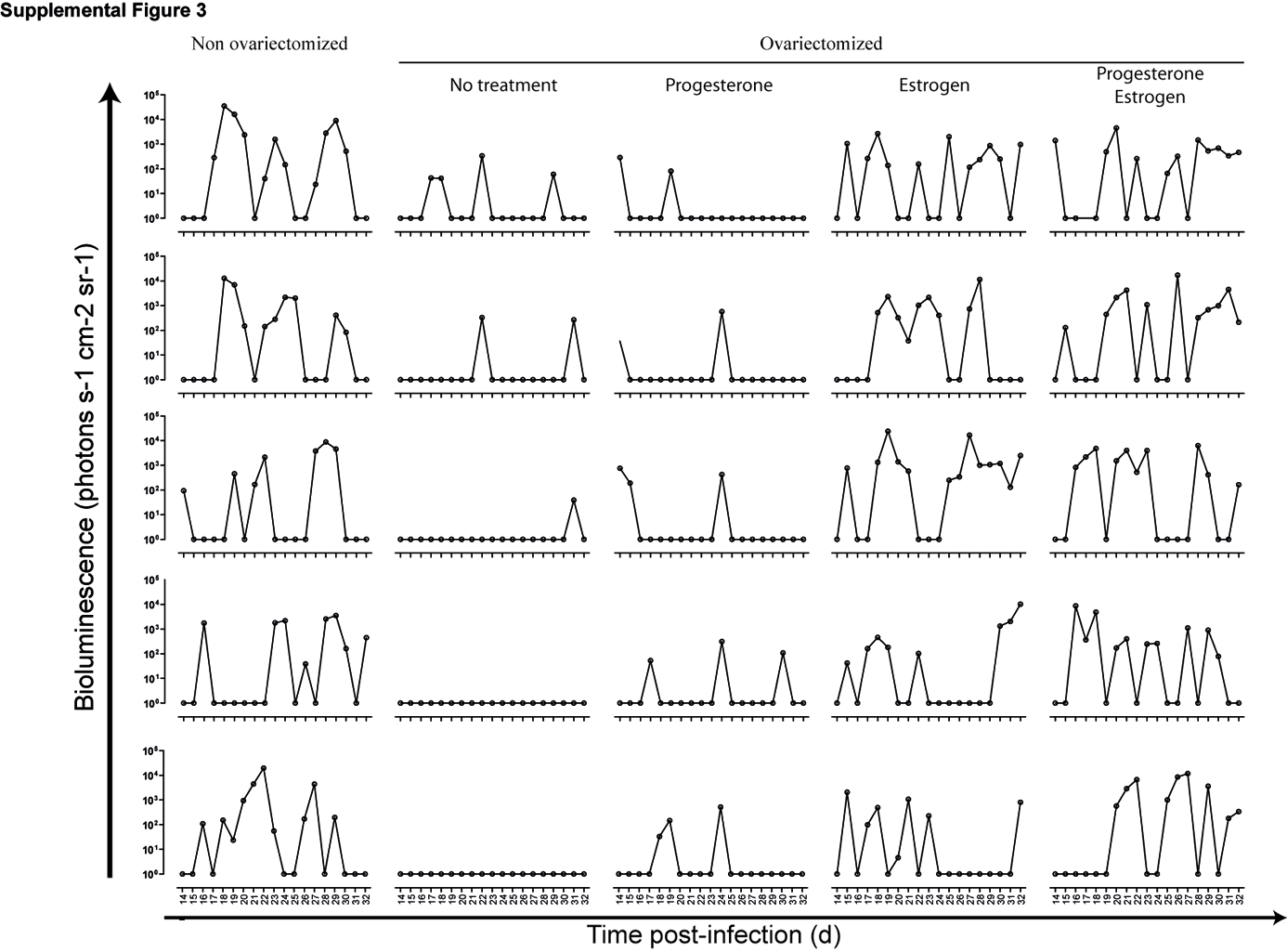

Supplement: Figure S3 — Influence of estrous cycle on genital MHV-68 excretion after intranasal infection. Control female mice and ovariectomized mice, implanted or not with slow-release hormonal pellets (progesterone and/or estrogen), were infected intranasally (104 PFU) with WT luciferase+ MHV-68 under general anaesthesia. Individual genital signals were monitored between days 14 and 32 post-infection. For the reliable comparison of signal intensities, the signal intensities were measured from equivalent regions of interest after subtraction of individual backgrounds measured daily in the right thoracic region. Each point shows one measurement. 5 individual mice per group are shown. (TIF) [file ppat.1003292.s003.tif]

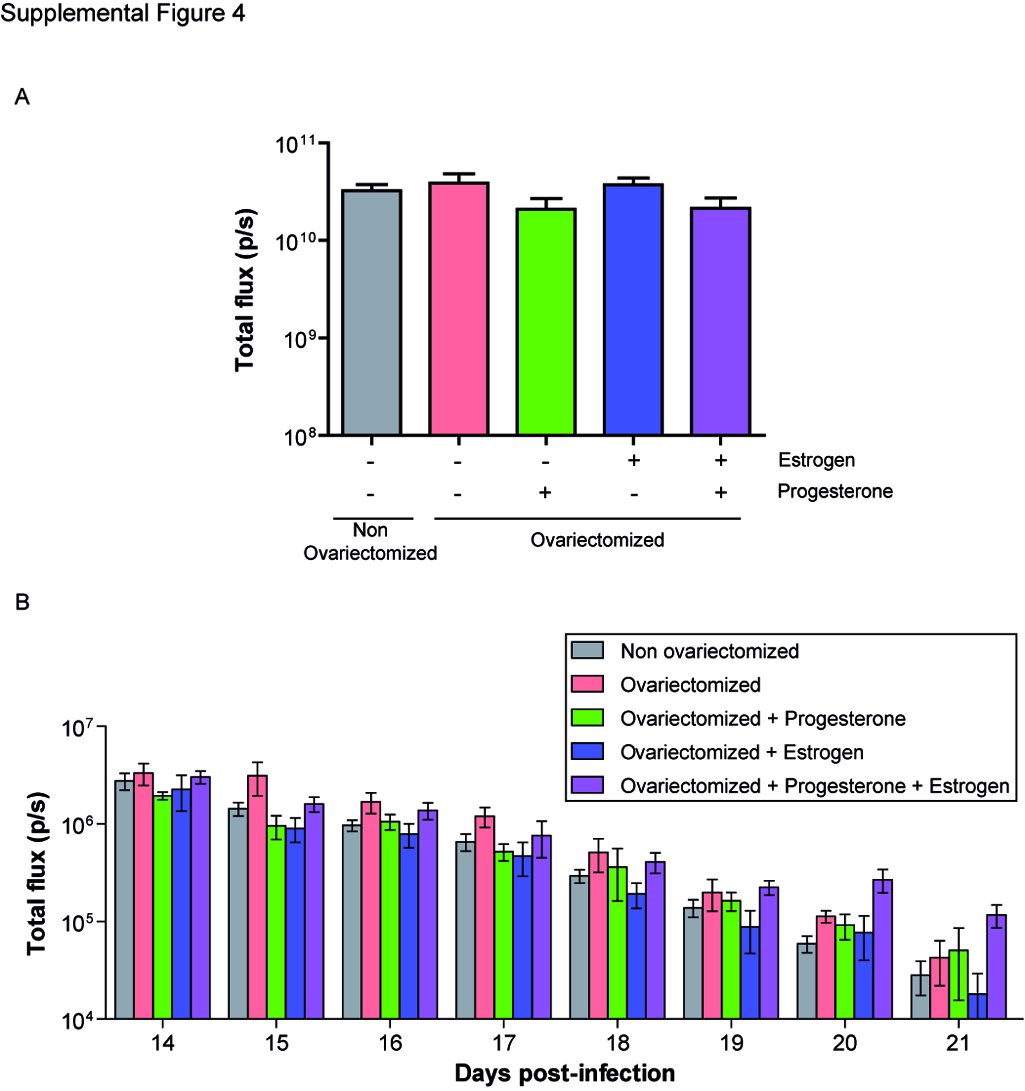

Supplement: Figure S4 — Influence of estrous cycle on lung and SCLN luciferase signals after intranasal infection. Control female mice and ovariectomized mice, implanted or not with slow-release hormonal pellets (progesterone and/or estrogen), were infected intranasally (104 PFU) with WT luciferase+ MHV-68 under general anaesthesia. A. Presence of lung signals was monitored at day 7 post-infection. The data presented are the average for triplicate measurements +/− standard error of the mean and were analyzed by 1way ANOVA and Bonferroni post-tests, no statistically significant difference was observed upon treatment. B. Lymphoid infection was monitored from day 14 to day 21 post-infection. The data presented are the average for triplicate measurements +/− standard error of the mean and were analyzed by 1way ANOVA and Bonferroni post-tests, no statistically significant difference was observed upon treatment. (TIF) [file ppat.1003292.s004.tif]

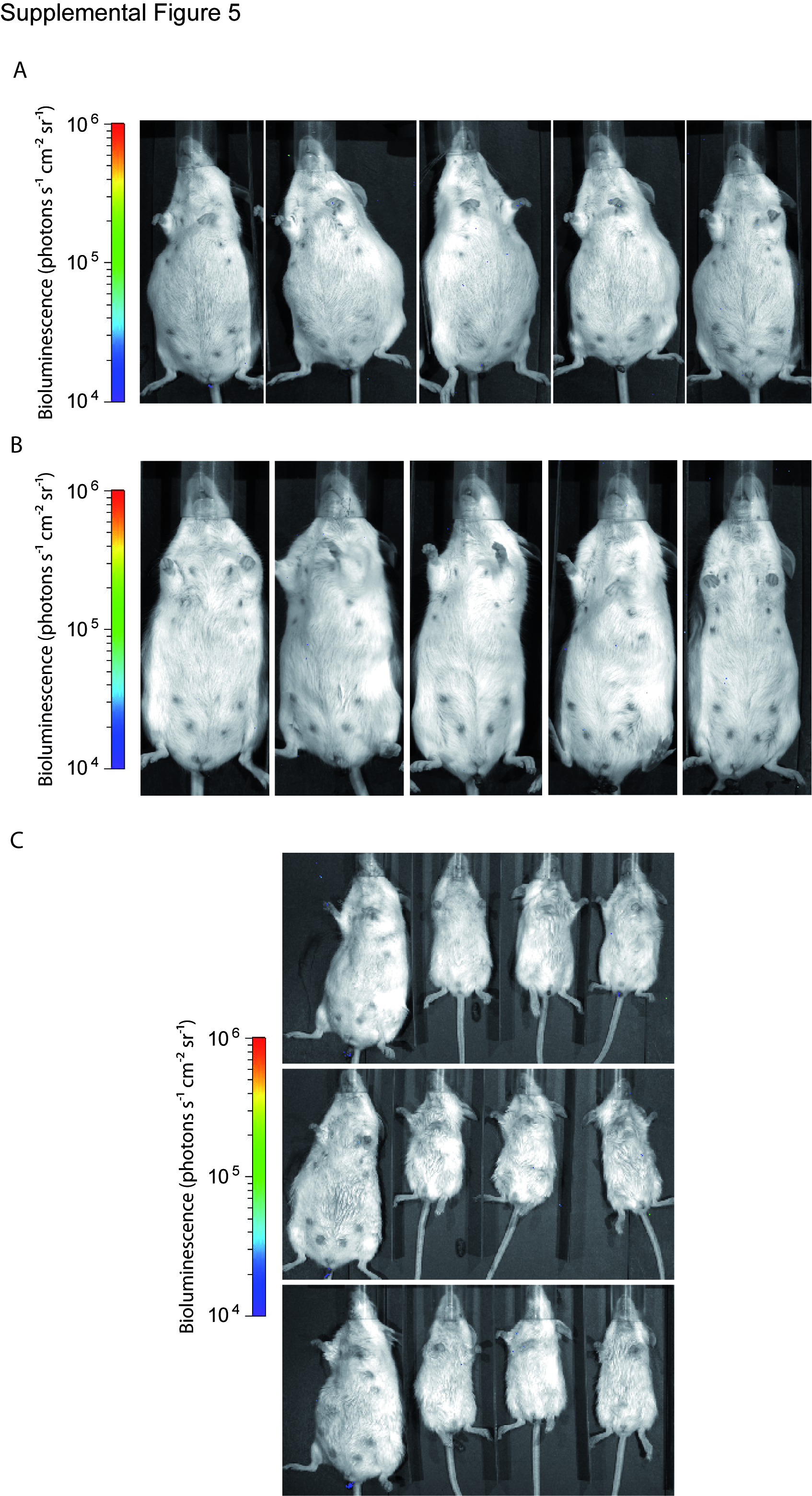

Supplement: Figure S5 — Luciferase signal in pregnant mice and their offspring. Female mice were infected intranasally (104 PFU) with WT luciferase+ MHV-68 under general anaesthesia, and then injected with luciferin and imaged every day. At the time of the first observation of genital signal, infected females were mated with uninfected males. A. Pregnant females were then injected with luciferin and imaged around day 20 post-mating. Images show 5 representative mice. B. The same mice were imaged similarly the day after delivery. Images show 5 representative mice. C. These mice and their litter were finally injected with luciferin and imaged 2 weeks post-delivery. Images show 3 representative mice with three of their pups. The scale bars (photons sec−1 cm−2 steradian−1) show the color scheme for signal intensity. (TIF) [file ppat.1003292.s005.tif]

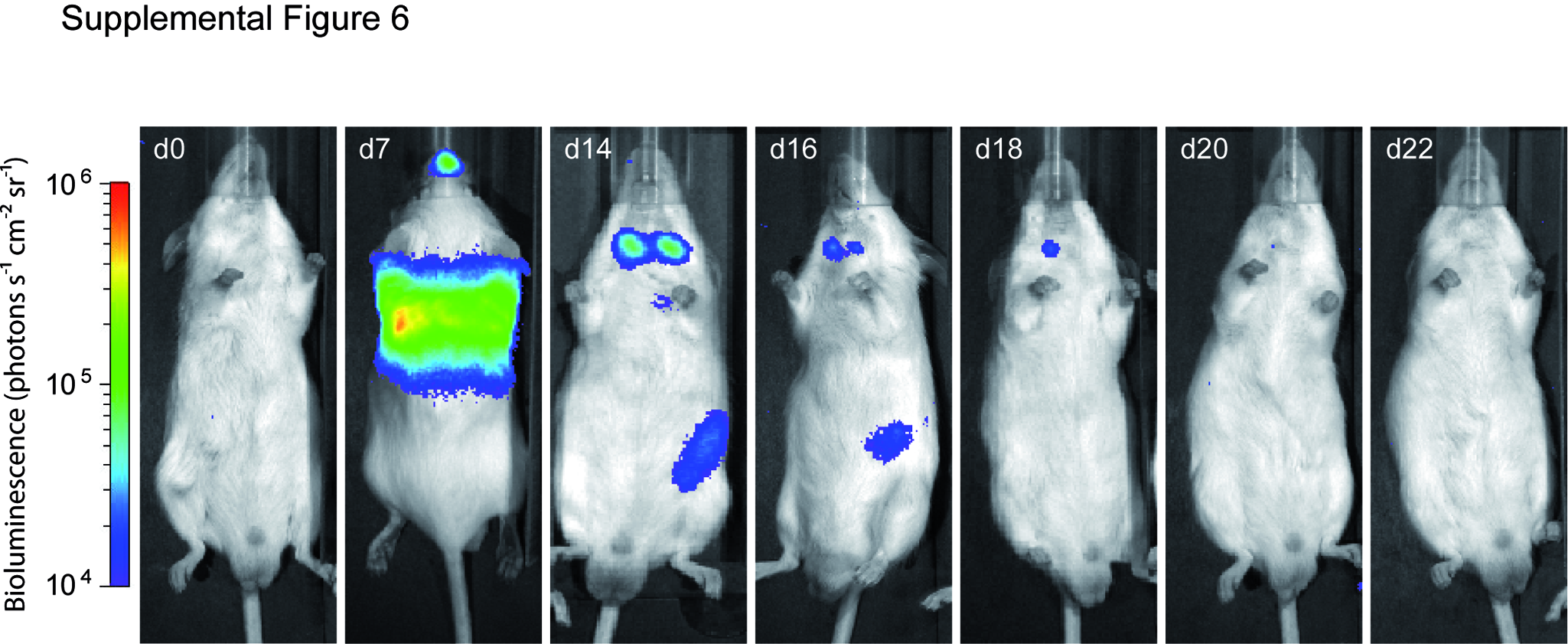

Supplement: Figure S6 — In vivo intranasal infection of males mice by luciferase-expressing MHV-68. 8 weeks-old male BALB/c mice (n = 10) were infected intranasally (104 PFU) with WT luciferase+ MHV-68 under general anaesthesia, and then injected with luciferin before in vivo imaging. Images show a representative mouse at days 0, 7, 14, 16, 18, 20 and 22 p.i.. The scale bar (photons sec−1 cm−2 steradian−1) shows the color scheme for signal intensity. (TIF) [file ppat.1003292.s006.tif]

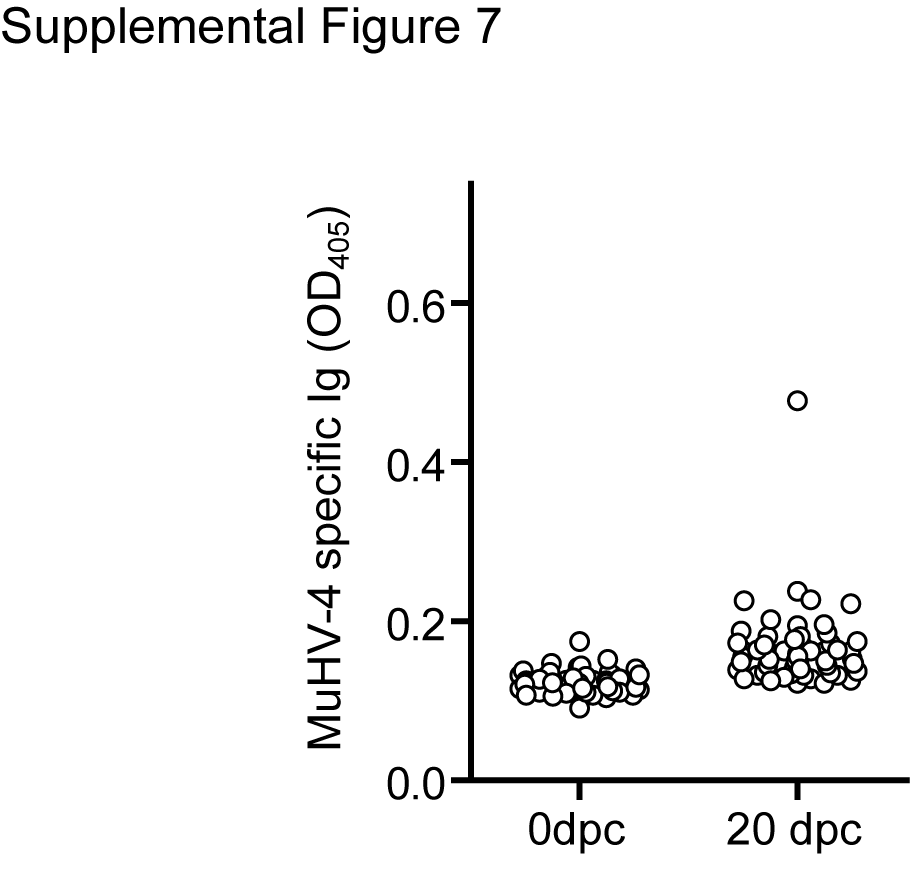

Supplement: Figure S7 — MHV-68 transmission from genitally infected males to naïve females. Genitally infected males (n = 13) (infected after contact with infected females excreting the virus in the genital tract) were mixed with uninfected females (at least 3 per male). MHV-68 infection of females was monitored 18 days post-contact by detection of anti-MHV-68 specific antibodies as described in the Material and Methods. (TIF) [file ppat.1003292.s007.tif]
